# Thioether-Containing Zirconium(Alkoxy)Siloxanes: Synthesis and Study of Dielectric and Mechanical Properties of Silica-Filled Polydimethylsiloxane Compositions Cured by Them

**DOI:** 10.3390/polym15163361

**Published:** 2023-08-10

**Authors:** Alexander N. Tarasenkov, Maria S. Parshina, Galina P. Goncharuk, Kirill M. Borisov, Evgeniy K. Golubev, Ivan B. Meshkov, Georgiy V. Cherkaev, Vitaliy G. Shevchenko, Sergey A. Ponomarenko, Aziz M. Muzafarov

**Affiliations:** 1 N. S. Enikolopov Institute of Synthetic Polymer Materials, Russian Academy of Sciences (ISPM RAS), Profsoyuznaya 70, 117393 Moscow, Russia; maria.parshina@list.ru (M.S.P.); ggoncharuk@ispm.ru (G.P.G.); borisov@ispm.ru (K.M.B.); golubev@ispm.ru (E.K.G.); ivanbm@ispm.ru (I.B.M.); cherkaevgv@ispm.ru (G.V.C.); shev@ispm.ru (V.G.S.); ponomarenko@ispm.ru (S.A.P.); aziz@ispm.ru (A.M.M.); 2 A. N. Nesmeyanov Institute of Organoelement Compounds, Russian Academy of Sciences (INEOS RAS), Vavilova 28, 119991 Moscow, Russia

**Keywords:** hydrothiolation, metalloalkoxysiloxanes, Rebrov’s salts, silicon composites, dielectric constant

## Abstract

A number of thioether-containing zirconium siloxanes, differing in their composition and metal atom shielding degree with a siloxy substituent, were synthesized and characterized. Synthesis of such compounds made it possible to evaluate the effect of sulfur atoms’ presence in the cured compositions on their dielectric properties, as well as to evaluate their curing ability and influence on mechanical characteristics compared to the sulfur-free analogs obtained earlier. Studying a wide range of compositions differing in their content and ratio of metallosiloxane and silica components revealed that such systems are still typical dielectrics. At the same time, the introduction of thioether groups can provide increased dielectric constant and conductivity in comparison with previously obtained sulfur-free similar compositions in the <10^2^ Hz frequency range (dielectric constant up to ~10–30 at frequency range 1–10 Hz). As before, the dielectric parameters increase is directly determined by the silica component proportion in the cured material. It is also shown that varying sulfur-containing zirconium siloxanes structure and functionality and its combination with previously obtained sulfur–free analogs, along with varying the functionality and rubber chain length, can be an effective tool for changing the dielectric and mechanical material parameters in a wide range (tensile strength 0.5–7 Mpa, elastic deformation 2–300%), which determine the prospects for the use of such cured systems as dielectric elastomers for various purposes.

## 1. Introduction

Dielectric elastomers are currently being used in motors, optical devices, sensors, energy storage, robotics, artificial muscles, etc. [1]. Dielectric elastomer is an electro-active polymer (EAP) capable of being polarized and changing its size and shape under the applied electric field and is the main functional element in such applications [2,3,4]. This performs by electrically driven mass transport of ions or electrically charged species in polymer [5]. The active study of the filled cross-linked systems behavior under various external conditions began in the 80s of the 20th centuries, and in the 1990s, the main approaches were already formulated, in particular, to the implementation of the concept of creating artificial muscles using dielectric elastomers [6,7]. Despite the many approaches developed, there is currently a surge of interest in creating new approaches for so-called “soft Actuators” based on EAPs.

Depending on the potential application area, EAP should have a number of required characteristics, the most important of which is the increased dielectric constant, mechanical strength (0.1–25 Mpa), and the ability to develop small or large reversible deformations while maintaining conductivity [8]. Among the polymers that can be used are, for example, fluoropolymers, polyacrylates, polyurethanes, and silicones [2,9,10,11]. Silicone elastomers have a number of features such as a wide range of mechanical properties regulation, a long exploitation period, a wide range of operating temperatures, biostability, formability [12], as well as high response speed and energy density [13], relatively low dielectric and mechanical losses [5]. This defines their use as dielectric elastomers in actuators. However, silicones have a relatively low dielectric constant [14], which necessitates the use of high voltage to activate the energy converter. The main approaches to increasing the dielectric permittivity of silicones are filler adding and chemical modification of the polymer chain.

Filling involves the introduction of conductive or high-dielectric fillers at the mixing stage. Such fillers can be complex inorganic salts [15,16], graphene particles, and carbon nanotubes [17,18], as well as metal oxides in the nano- or micro-sized form [19,20,21]. One of the most common approaches is the introduction of nano- and micro-sized silica into silicone material which has not only a good affinity for silicone but also increases and stabilizes the dielectric characteristics and strengthens the material, and €s capable of modification to give good compatibility with polymer matrix and desired properties to the final material [22,23,24,25]. Filling allows not only to increase the dielectric constant and conductivity but also strengthens the material. However, there is a problem with filler distribution over the material volume in this approach.

Chemical modification of the polymer matrix or initial monomer eliminates the problem of distribution of the functional filler in the material. Thus, it is possible to introduce polar groups of various natures into the system [26,27,28,29], as well as to obtain ionomer materials with high dielectric properties [30]. The literature reports analyses show that the introduction of polar thioether groups into polymer structure can increase its dielectric constant. It can be achieved through one of the most convenient methods of click chemistry—hydrothiolation of vinyl containing silicone polymer and precursors. The availability of raw materials and a wide range of thiols opens up wide possibilities for fast modification and gives the desired functionality to the polymer matrix [31]. In this way, it is also possible to form an EAP network, as well as to control not only the dielectric but also the physicomechanical properties of the polymer matrix [19,32]. It was shown that even in the presence of nonpolar hydrocarbon substituents in combination with a thioether group, it is possible to increase the dielectric constant values of siloxane elastomers [33]. This method also has its drawbacks, which mainly consist of problems with the compatibility of the components when their physicochemical properties change due to modification.

We have previously demonstrated the approach of the silicon rubber cross-linking in the presence of SiO_2_ precursor by using functional metallosiloxanes, providing SiO_2_-filled silicone elastomeric material with higher values of dielectric permittivity compared to commercial silicone compounds [34]. In our case, one of the ways to introduce sulfur atoms into the system is to obtain thioether-containing metallosiloxanes. This allows the heteroatoms to be evenly distributed in combination with the in situ formation of a silica phase over the material volume due to the catalytic effect of the central metallosiloxane atom, potentially increasing its dielectric properties. This work deals with the study of the possibility of obtaining such metallosiloxanes and the effect of the sulfur atoms introduction on the dielectric properties of the final material.

## 2. Materials and Methods

### 2.1. Characterization Methods

^1^H and ^29^Si NMR spectra were recorded at room temperature on Bruker Avance 300 and Bruker Avance 400 NMR spectrometers (Mannheim, Germany) using the standard pulse sequences of the Bruker software (TopSpin^TM^). The spectra were processed in MestReNova software (v. 12.0). The solvent residual signals—CHCl_3_ were used as internal standards (δ 7.25 ppm).

Elemental analysis was carried out on a Carlo Erba 1106 instrument (Milano, Italy). The relative error of determination of the contents of silicon, carbon, and hydrogen (%wt.) did not exceed 0.1%. The silicon, carbon, and hydrogen contents were determined by burning a sample (5–10 × 10^–3^ g) in an oxygen gas atmosphere at 950 °C. The averaged data on two dimensions are presented.

The real and imaginary parts of permittivity, conductivity, modulus, and the loss factor were measured using an impedance analyzer Novocontrol Alpha-A (Installation Concept 40) and the dielectric cell ZGS Alpha Active Sample Cell with gold disk electrodes with a diameter of 20 mm (Montabaur, Germany). Measurements were performed in the frequency range of 10^−1^–10^6^ Hz at room temperature.

The mechanical properties of the compositions were determined in the uniaxial extension mode on the universal testing machine Autograph AGS-H by Shimadzu (Kyoto, Japan). The samples were strips with a working part size of 3 × 20 mm; the rate of extension was 10 mm/min. The tensile strength and elongation at break were measured. The averaged data on three-five tests are presented.

WAXS diffraction patterns for the studied samples were recorded on a D8 Advance diffractometer (Bruker AXS, Mannheim, Germany) with a focusing germanium crystal monochromator on the primary beam (CuKα1 radiation, wavelength λ = 0.1541 nm) in “transmission” mode in the range of scattering angles 2θ = 10°–40° at room temperature.

IR spectra were recorded on a Bruker Tensor 27 spectrometer (Germany) in the ATR mode of 4 scans for each wave number in the range of 550–4000 cm^−1^.

Scanning electron microscopy (SEM) was performed using a JCM-6000 PLUS microscope (Tokyo, Japan) equipped with an energy-dispersive spectrometer at accelerating voltages of 5–15 kV. The samples were subjected to gold dusting before testing.

### 2.2. Initial Materials

To prepare the compositions, commercially available polydimethylsiloxane (PDMS) rubbers (hereinafter PDMS-A, G, and E) of different molecular weights were used. The products are characterized by small molecular weight and dynamic viscosity values: PDMS-A—M_W_~20,000, 1.5–2 Pa·s; PDMS-G—M_W_~55,000, 5–9 Pa·s; and PDMS-E—M_W_~120,000, 80–120 Pa·s. The following reagents were used without further purification: 3-mercaptopropylmethyldimethoxysilane (95%, “abcr”), 3-mercaptopropyltrimethoxysilane (95%, “abcr”), ZrCl_4_ (≥99.5%, “Lanhit”). Vinyldimethylmethoxysilane, vinyltrimethoxysilane, and vinyltriethoxysilane were distilled under argon prior to use. Toluene, MTBE, THF, and ethanol were dried by prolonged boiling, followed domain by distillation over CaH_2_ under argon, and stored over 3 Å molecular sieves.

### 2.3. Composition Components Preparation

#### 2.3.1. PDMS and PEOS Preparation

PDMS rubbers pre-blocked with 3-aminopropyltriethoxysilane were obtained according to the procedure [35].

Hyperbranched polyethoxysiloxane (PEOS) was prepared according to the procedure [35]. M_w_~1600, M_w_/M_n_~4.1 (PSS).

#### 2.3.2. Thioether-Containing Silanes Preparation

Silane 1. All operations were carried out in an argon atmosphere. A mixture of vinyldimethylmethoxysilane (3.37 g, 0.0290 mol) and 3-mercaptopropyl(methyl)dimethoxysilane (4.76 g, 0.0264 mol) in 35 mL of dried MTBE was irradiated with a UV lamp (365 nm) under stirring at room temperature for 5 h. Reaction completeness was determined by the disappearance of mercapto group proton signals at δ 1.32 ppm in the ^1^H NMR spectrum. The mixture obtained was evaporated from volatiles in a vacuum (1 Torr), resulting in a low-viscosity transparent, slightly yellowish liquid. The yield of the product was 7.75 g (99%). ^1^H NMR (CDCl_3_, δ ppm): 0.10, 0.12 (both s, 9H, SiCH_3_); 0.66–0.77, 0.88–0.99 (both m, 4H, SiCH_2_); 1.55–1.72 (m, 2H, CH_2_); 2.47–2.63 (m, 4H, SCH_2_), 3.42, 3.49 (both s, 9H, OCH_3_). ^29^Si NMR (CDCl_3_, δ ppm): −1.6, 6.6, 18.0.

Silane 2. All operations were carried out in an argon atmosphere. A mixture of vinyltrimethoxysilane (3.19 g, 0.022 mol) and 3-mercaptopropyl(methyl)dimethoxysilane (3.43 g, 0.019 mol) was irradiated with a UV lamp (365 nm) under stirring and at room temperature for 30 min. Reaction completeness was determined by the disappearance of mercapto group proton signals at δ 1.32 ppm in the ^1^H NMR spectrum. The mixture obtained was evaporated from volatiles in a vacuum (1 Torr), resulting in a low-viscosity transparent colorless liquid. The yield of the product was 7.14 g (99%). ^1^H NMR (CDCl_3_, δ ppm): 0.10 (s, 3H, SiCH_3_); 0.65–0.77, 0.92–1.03 (both m, 4H, SiCH_2_); 1.56–1.71 (m, 2H, CH_2_); 2.47–2.66 (m, 4H, SCH_2_); 3.49, 3.56 (both s, 15H, OCH_3_). 

Silane 3. All operations were carried out in an argon atmosphere. A mixture of vinyltrimethoxysilane (8.71 g, 0.0590 mol) and 3-mercaptopropyltrimethoxysilane (11.53 g, 0.0590 mol) was irradiated with a UV lamp (365 nm) under stirring and at room temperature for 2 h. Reaction completeness was determined by the disappearance of mercapto, and vinyl group proton signals at δ 1.32 and ~6.0 ppm in the ^1^H NMR spectrum, accordingly. The product was obtained in the form of low-viscosity transparent colorless liquid with a quantitative yield (20.64 g). ^1^H NMR (CDCl_3_; δ ppm, J Hz): 0.74 (t, 2H, SiCH_2_ propyl, J = 8.2); 0.92–1.04 (m, 2H, SiCH_2_ ethyl); 1.68 (quint, 2H, CH_2_, J = 7.9); 2.48–2.65 (m, 4H, SCH_2_); 3.55, 3.56 (both s, 18H, OCH_3_).

Silane 4 was obtained similarly to *3* from vinyltriethoxysilane (10.91 g, 0.0573 mol) and 3-mercaptopropyltrimethoxysilane (11.26 g, 0.0573 mol). The product was obtained in the form of low-viscosity transparent colorless liquid with a quantitative yield (22.83 g). ^1^H NMR (CDCl_3_; δ ppm, J Hz): 0.72 (t, 2H, SiCH_2_ propyl, J = 8.5); 0.95 (t, 2H, SiCH_2_ ethyl, J = 8.6); 1.19 (t, 9H, CH_3_, J = 7.3); 1.67 (quint, 2H, CH_2_, J = 7.9); 2.52 (t, 2H, SCH_2_, J = 7.2); 2.58 (t, 2H, SCH_2_, J = 8.8); 3.53 (s, 9H, OCH_3_); 3.80 (quart, 6H, OCH_2_, J = 7.3).

Ethylundecenoate. Solution of NaOH (4.78 g, 0.1195 mol) in 10 mL of water was added to the undecenoic acid (22.01 g, 0.1194 mol) solution in 50 mL of acetone under stirring. Heating of the mixture and precipitation over the entire volume was observed. The mixture was then stirred until completely cooled to room temperature. The main amount of water was then removed with acetone several times. 100 mL of ethanol was added to the residue, and then trimethylchlorosilane (4.52 g, 0.1410 mol) was quickly added to the resulting suspension under stirring. The resulting mixture was refluxed for 4 h and filtered from the precipitate. The solution was washed with NaCl water (with NaCl) until the neutral reaction. The organic layer was kept over anhydrous Na_2_SO_4_ for a day and then filtered and evaporated from volatiles (1 Torr, 60 °C), resulting in low-viscosity transparent yellow liquid. The yield of the product was 23.49 g (89%). ^1^H NMR (CDCl_3_; δ ppm, J Hz): 1.14–1.44, 1.49–1.71 (both m, 15H, CH_2_, CH_3_); 2.02 (quart, 2H, CH_2_CH=CH_2_, J = 6.9); 2.26 (t, 2H, CH_2_C(O), J = 7.4); 4.11 (quart, 2H, OCH_2_, J = 7.0); 4.85–5.05 (m, 2H, =CH_2_); 5.67–5.90 (m, 1H, =CH).

Silane 5. All operations were carried out in an argon atmosphermethyl undecenoate yl undecenoate (3.44 g, 0.0162 mol), and 3-mercaptopropyltrimethoxysilane (3.18 g, 0.0162 mol) was irradiated with a UV lamp (365 nm) under stirring at room temperature for 4 h. Reaction completeness was determined by disappearance of the mercapto group and double bond protons signals at δ 1.32 and 4.90, 5.82 ppm in ^1^H NMR spectrum accordingly. The product was obtained in the form of low-viscosity transparent yellow liquid with a quantitative yield (6.22 g). ^1^H NMR (CDCl_3_; δ ppm, J Hz): 0.73 (t, 2H, SiCH_2_, J = 8.3); 1.15–1.40, 1.42–1.76 (both m, 21H, CH_2_, CH_3_); 2.25 (t, 2H, CH_2_C(O), J = 7.3); 2.48 (quart, 4H, SCH_2_, J = 7.9); 3.54 (s, 9H, OCH_3_); 4.09 (quart, 2H, OCH_2_, J = 7.1).

#### 2.3.3. Thioether-Containing Zr-Siloxanes Preparation

General procedure for thioether-containing sodium alkoxy silanolates obtaining (R_3_SiONa). All operations were carried out in an argon atmosphere. NaOH was added to the solution of thioester-containing alkoxysilane in the dried THF at [NaOH]:[silane] = 1:1 molar ratio. The mixture was refluxed until sodium hydroxide was completely dissolved, after which volatiles was removed (1 Torr, 60 °C), and the residue was then used for the synthesis.

General procedure for thioether-containing zirconium siloxanes obtaining. All operations were carried out in an argon atmosphere. Silanolate solution in dried toluene was added to the suspension of zirconium (IV) chloride in dried toluene. In the case of non-fully siloxy substituted products, added mixture additionally contained the required amount of sodium solution in ethanol (according to the method [36]) at [Zr]:[oNa] = 1:4 molar ratio. The resulting mixture was then stirred at 50 °C till a neutral medium product solution was then separated from the precipitate (NaCl) by centrifugation (9000 rpm, 30 min, 18 °C). In the case of non-fully siloxy substituted products, dried toluene was additionally added to the mother solution for subsequent ethanol removal from the mixture with azeotrope. The yield of the product was determined by its content in the aliquot of the final solution.

ZrS1(4-0) was obtained by the interaction of a suspension of zirconium (IV) chloride (0.58 g, 0.0025 mol) in 10 mL of dried toluene with a SiONa1 (2.98 g, 0.0098 mol) solution in 20 mL of dried toluene for 3 h. The dry product was a yellow opalescent non-flowing mass. The yield of the product was 2.79 g (92%). Found (%): C, 38.35; H, 8.11; Si, 18.16; S, 10.24. C_40_H_100_O_12_S_4_Si_8_Zr. Calculated (%): C, 39.46; H, 8.28; Si, 18.46; S, 10.54. SiONa1 was obtained by the interaction of NaOH (0.40 g, 0.0100 mol) with a solution of 1 (2.96 g, 0.0100 mol) in 10 mL of dried THF. The mixture was refluxed for 1.5 h. After volatiles removing the product was obtained in the form of a yellow waxy mass. The product yield was 2.98 g (98%).

ZrS2(4-0) was obtained by the interaction of a suspension of zirconium (IV) chloride (1.15 g, 0.0049 mol) in 10 mL of dried toluene with a SiONa2 (6.62 g, 0.0197 mol) solution in 20 mL of dried toluene for 2 h. The dry product was a yellow opalescent non-flowing mass. The yield of the product was 4.81 g (73%). Found (%): C, 35.52; H, 7.60; Si, 17.04; S, 9.87. C_40_H_100_O_20_S_4_Si_8_Zr. Calculated (%): C, 35.71; H, 7.49; Si, 16.70; S, 9.53. SiONa2 was obtained by the interaction of NaOH (0.81 g, 0.0203 mol) with a solution of *2* (6.67 g, 0.0203 mol) in 20 mL of dried THF. The mixture was refluxed for 2 h. After volatiles removing the product was obtained in the form of a yellow waxy mass. The product yield was 6.62 g (97%).

ZrS3(4-0) was obtained by the interaction of a suspension of zirconium (IV) chloride (1.86 g, 0.0080 mol) in 15 mL of dried toluene with a SiONa3 (11.26 g, 0.0320 mol) solution in 80 mL of dried toluene for 3 h. The dry product was a yellowish opalescent, highly viscous liquid. The yield of the product was 7.58 g (67%). Found (%): C, 34.30; H, 7.25; Si, 16.05; S, 9.28. C_40_H_100_O_24_S_4_Si_8_Zr. Calculated (%): C, 34.09; H, 7.15; Si, 15.94; S, 9.10. SiONa3 was obtained by the interaction of NaOH (1.32 g, 0.0331 mol) with a solution of *3* (11.39 g, 0.0331 mol) in 40 mL of dried THF. The mixture was refluxed for 2 h. After volatiles removing the product was obtained in the form of a yellow waxy mass. The product yield was 11.26 g (97%).

ZrS4(4-0) was obtained by the interaction of a suspension of zirconium (IV) chloride (1.53 g, 0.0066 mol) in 20 mL of dried toluene with a SiONa4 (10.65 g, 0.0272 mol) solution in 90 mL of dried toluene for 3 h. The dry product was a yellow opalescent, highly viscous liquid. The yield of the product was 9.66 g (93%). Found (%): C, 37.91; H, 7.78; Si, 15.27; S, 8.68. C_52_H_124_O_24_S_4_Si_8_Zr. Calculated (%): C, 39.59; H, 7.92; Si, 14.24; S, 8.13. SiONa4 was obtained by the interaction of NaOH (1.05 g, 0.0263 mol) with a solution of 4 (10.18 g, 0.0263 mol) in 40 mL of dried THF. The mixture was refluxed for 1.5 h. After volatiles removing the product was obtained in the form of a yellow-orange waxy mass. The product yield was 10.65 g (>99%).

ZrS4(2-2) was obtained by the interaction of a suspension of zirconium (IV) chloride (1.75 g, 0.0075 mol) in 20 mL of dried toluene with a mixture of SiONa4 (5.92 g, 0.0150 mol) solution in 40 mL of dried toluene and sodium (0.35 g, 0.0150 mol) solution in 12 mL of dried ethanol for 3 h. After separation from the precipitate, 80 mL of dried toluene was added, and then 100 mL of solvent (1 Torr, 45 °C) was removed from the solution. A clear yellowish final solution was obtained. The dry product was a yellow transparent, highly viscous liquid. The yield of the product was 6.81 g (98%). ^1^H NMR (CDCl_3_; δ ppm): 0.65–0.80 (m, 4H, SiCH_2_ propyl); 0.91–1.05 (m, 4H, SiCH_2_ ethyl); 1.12–1.33 (m, 36H, CH_3_, OCH_3_); 1.60–1.79 (m, 4H, CH_2_); 2.46–2.72 (m, 8H, SCH_2_); 3.44–3.93 (m, 16H, OCH_2_). Found (%): C, 39.06; H, 7.96; Si, 12.41; S, 7.09. C_30_H_72_O_14_S_2_Si_4_Zr. Calculated (%): C, 38.97; H, 7.85; Si, 12.15; S, 6.94. SiONa4 was obtained by the interaction of NaOH (0.60 g, 0.0150 mol) with a solution of 4 (5.80 g, 0.0150 mol) in 40 mL of dried THF.

ZrS4(1-3) was obtained by the interaction of a suspension of zirconium (IV) chloride (3.29 g, 0.0141 mol) in 30 mL of dried toluene with a mixture of SiONa4 (5.64 g, 0.0144 mol) solution in 60 mL of dried toluene and sodium (0.97 g, 0.0423 mol) solution in 25 mL of dried ethanol for 3 h. After separation from the precipitate, 100 mL of dried toluene was added, and then 100 mL of solvent (1 Torr, 45 °C) was removed from the solution. A clear yellowish final solution was obtained. The dry product was a yellowish transparent, highly non-flowing liquid. The yield of the product was 8.21 g (97%). Found (%): C, 38.13; H, 7.60; Si, 9.62; Zr, 15.06. C_19_H_46_O_9_sSi_2_Zr. Calculated (%): C, 38.16; H, 7.75; Si, 9.39; Zr, 15.25. SiONa4 was obtained by the interaction of NaOH (0.56 g, 0.0141 mol) with a solution of 4 (5.45 g, 0.0141 mol) in 35 mL of dried THF. 

ZrSU(2-2) was obtained by the interaction of a suspension of zirconium (IV) chloride (1.89 g, 0.0081 mol) in 10 mL of dried toluene with a mixture of SiONa5 (6.65 g, 0.0160 mol) solution in 75 mL of dried toluene and sodium (0.37 g, 0.0162 mol) solution in 14 mL of dried ethanol for 3 h. After separation from the precipitate, 80 mL of dried toluene was added, and then 150 mL of solvent (1 Torr, 45 °C) was removed from the solution. A clear yellow-orange final solution was obtained. The dry product was an orange transparent, highly viscous liquid. The yield of the product was 6.93 g (88%). ^1^H NMR (CDCl_3_; δ ppm, J Hz): 0.51–0.88 (broad peak, 4H, SiCH_2_); 1.12–1.43, 1.44–1.82 (both m, 60H, CH_2_, CH_3_, OCH_3_); 2.27 (t, 4H, CH_2_C(O), J = 7.8); 2.38–2.60 (m, 8H, SCH_2_); 3.44–3.87 (m, 4H, ZrOCH_2_); 4.10 (quart, 4H, COCH_2_, J = 7.3). Found (%): C, 49.74; H, 8.68; Si, 5.82; S, 6.88. C_40_H_84_O_12_S_2_Si_2_Zr. Calculated (%): C, 49.60; H, 8.74; Si, 5.80; S, 6.62. SiONa5 was obtained by the interaction of NaOH (0.65 g, 0.0162 mol) with a solution of 5 (6.62 g, 0.0162 mol) in 50 mL of dried THF. The mixture was refluxed for 1.5 h. After volatiles removing the product was obtained in the form of a yellow-orange transparent non-flowing mass. The product yield was 6.65 g (99%).

#### 2.3.4. General Procedure for Preparation of Silicon Compositions

A toluene solution of initial components (PDMS, PEOS, and metallosiloxane) was poured onto a Teflon^®^ substrate and then kept at room temperature until most volatiles were evaporated. After that residual was heated following the temperature steps: 1 h—50 °C, 1 h—70 °C, 1 h—100 °C and 2 h—150 °C. The compositions were cooled, removed from the substrate, and tested.

## 3. Results

In our previous report, we investigated the dielectric properties of siloxane elastomer materials cured with methyl-, phenyl-, and vinyl silyl derivatives of metalloalkoxysiloxanes (MSs). In this work, we investigated the properties of compositions cured with thioether-containing MSs. Since zirconium siloxanes showed better vulcanizing properties and, as a result, more acceptable mechanical and dielectric properties, we focused here on the zirconium siloxane synthesis and using them for the preparation and further study of cured compositions based on PDMS.

**Figure 1:**
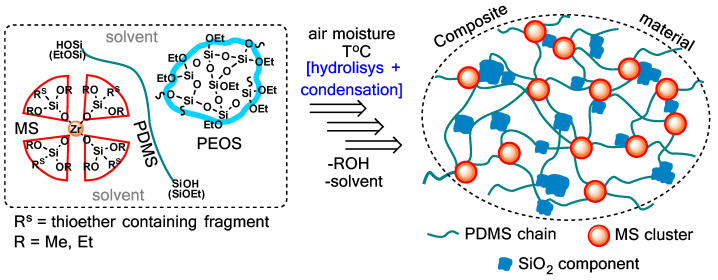
Scheme of formation of a three-component composite material.

As before, the objects for the study were obtained in the form of two-(MS + PDMS) and three-component (MS + PDMS + PEOS) compositions. Branched polyethoxysiloxane (PEOS) was introduced into the system to form a silica component inside the cured material during its condensation (Figure 1). As was shown earlier, its presence is critically important for increasing the permittivity in the low-frequency region [34].

**Figure 2:**
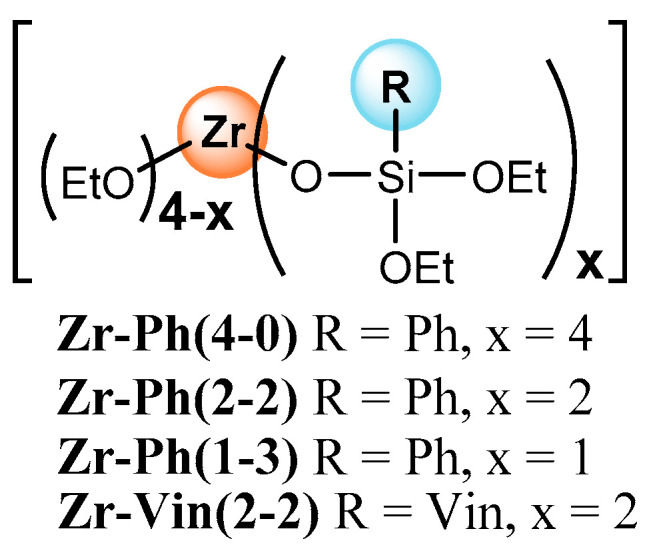
Previously obtained zirconium siloxanes.

The compositions for the study were obtained in the form of films with a thickness of 0.35 ± 0.03 mm. We used low molecular weight rubber of the “CKTH” trademark (hereinafter PDMS-A, G, and E, respectively), differing in molecular weight and functionality. As before, MS and PEOS amounts varied relative to the rubber weight, which was taken as a basis and remained 3 wt.pt. PEOS/MS initial ratio in the kneaded compositions varied from 0/2 to 6/1 wt.pt., depending on the MS activity and the quality of the resulting compositions. This approach is determined by the possibility of comparing the properties of newly obtained compositions with previous materials. The mechanical characteristics of the films were evaluated in terms of their tensile strength (maximum stress (σ) and elongation (ε) at the moment of film rupture). In the aspect of electrical properties, the comparison objects in work were both analogous compositions previously obtained using phenyl silyl and vinyl silyl derivatives of zirconium siloxanes [34,37,38] (Figure 2) and a film derived from commercially available two-component cured silicone. Table 1, Table 2 and Table 3 contain data on the electrical and mechanical properties of the compositions.

### 3.1. Compositions Cured with Methoxy Silyl Derivatives of Sulfur-Containing Zr-Siloxanes

At the first stage, a number of full siloxy substituted thioether containing methoxy silyl derivatives of Zr-siloxanes (ZrS1-3(4-0)) were obtained, differing in the number of methoxy groups in their structure. This approach made it possible to assess the influence of the organic component and the cross-linking degree on the quality of the formed material.

**Figure 3:**
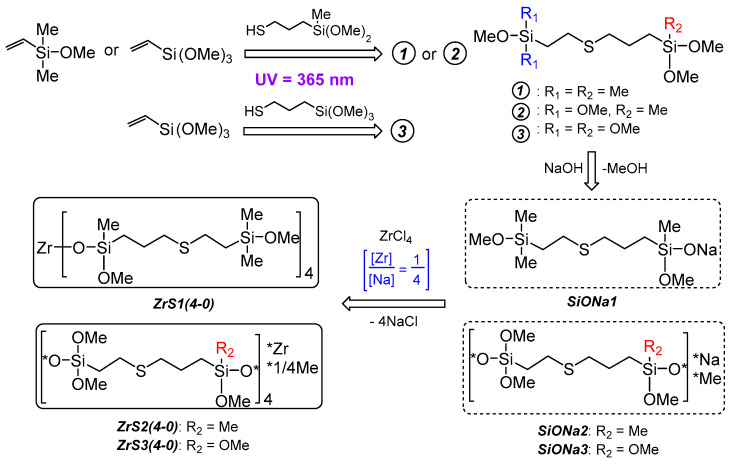
Scheme of ZrS1(4-0), ZrS2(4-0) and ZrS3(4-0) synthesis.

Such approach implementation was through the hydrothiolation reaction between vinylmethoxysilanes and 3-mercaptopropyl(methyl)dimethoxy- and 3-mercaptopropyltrimethoxysilane under UV irradiation (365 nm) thioester derivatives of silanes 1-3. Further interaction of 1-3 with NaOH in the boiling MTBE resulted in the corresponding silanolates—Rebrov’s salts (SiONa1-3) with quantitative yields. The synthesis of Zr-siloxanes was carried out by direct interaction of zirconium (IV) chloride with SiONa1-3 in a toluene medium at a molar ratio [Zr]:[Na] = 1:4 with MSs ZrS1-3(4-0) obtaining respectively (Figure 3). Thus, the synthetic relationship is silane x→SiONax→ZrSx(4-0), where x = 1, 2, 3. There is the most probable ZrS1(4-0) structure is presented based on possible steric difficulties and the activity of alkoxy groups. In the case of ZrS2(4-0) and ZrS3(4-0), it is difficult to talk about the preferred product of the “silane—alkali” interaction because the activity of the methoxy groups at both silicon atoms is sufficient for the reaction, and the study of sodium salts by ^1^H NMR methods indicates rather the formation of a products mixture (see Figure S1a in Supplementary Materials).

The synthesis of ZrS1(4-0), ZrS2(4-0), and ZrS3(4-0) was carried out in toluene, with a noticeable decrease in the product yield in the ZrS1(4-0)→ ZrS2(4-0)→ZrS3(4-0) series, presumably due to a decrease in the organic methyl silyl substituents proportion in the MS structure. Nevertheless, even in the case of ZrS3(4-0), the yield is quite high and can be further regulated by changing the concentration of the reaction mixture. As before, due to the high reactivity in the presence of air moisture, MS was stored and used in the form of dilute solutions (~15% by weight) under an inert layer without air access. Chromatographic analysis of such compounds is not possible, and their identification was carried out using elemental analysis. The elemental composition of all the products obtained is in good agreement with the calculated content of the elements. Dry products were non-volatile yellow transparent substances. 

**Figure 4:**
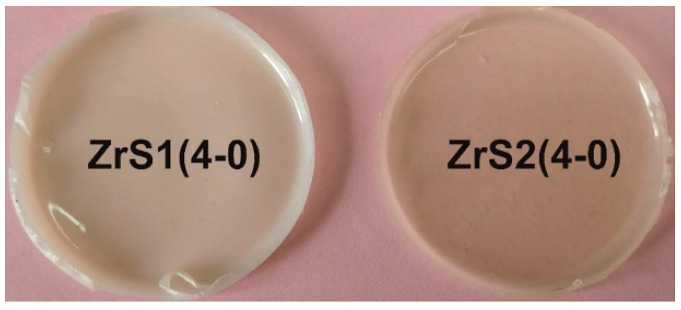
Compositions photos with the initial ratio ZrS(4-0)/PDMS-E*/PEOS = 1/3/2 wt.pt.

Table 1 contains data on the dielectric and mechanical characteristics of systems cured by MSs ZrS1-3(4-0). PDMS-E was used as a rubber in this case. It has appeared that the MSs do not work well as hardeners in the case of silanol rubber use, which is expressed in the poor quality of the final material: the films are heterogeneous and waxy. Acceptable quality films were obtained only when using PDMS-E* rubber, pre-blocked with 3-aminopropyltrimethoxysilane, i.e., with modified functionality. Such an effect can be associated presumably with the altered catalytic activity of the zirconium atom due to its shielding by bulk siloxy substituents and, as a consequence, different kinetics of the PDMS grid and the three-dimensional grid of filler and hardener formation. Thus, in order to obtain a homogeneous elastomeric material, in this case, the entire initial system must be ethoxy silyl-functional to equalize the rates of grid formation. At the same time, the use of ZrS1(4-0) with the largest proportion of methyl silyl substituents lead to a poorer components combination even in the case of modified rubber, which is not observed in the case of ZrS2(4-0) and ZrS3(4-0) (Figure 4).

**Figure 5:**
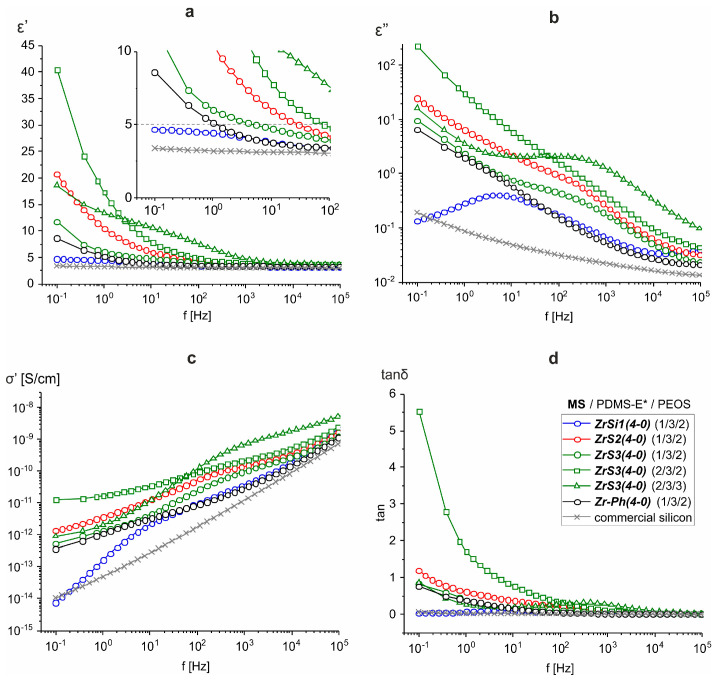
Frequency dependences of dielectric constant ε′ (**a**), dielectric losses ε″ (**b**), conductivity σ′ (**c**), and dielectric loss tangent tanδ (**d**) for three-component compositions ZrSx(4-0)/PDMS-E/PEOS at different initial ratios.

In terms of electrical properties, the same trend can be traced, as was observed in the case of other zirconium siloxane derivatives [34] (Figure 5). A sharp increase in the permittivity (ε′) is observed in the low-frequency region (f < 10 Hz), and such an effect is noticeable both in the MS fraction and initial PEOS/MS ratio increase, i.e., when the fraction of the silica component precursor is higher. And if the two-component system (Table 1, #13) is close in terms of its indicators to industrial silicone compounds (Table 1, #22), then permittivity can reach values of ε′ > 5 in the frequency range f < 100 Hz (Table 1, #15, 18) with an increase in silica filling. At the same time, the obtained permittivity values are slightly higher compared to similar films cured, in particular, with completely siloxy-substituted phenyl zirconium siloxane Zr-Ph(4-0) (Table 1, #20). Moreover, the difference increases when comparing values obtained with modified rubber (Table 1, #21). This indicates the effectiveness of introducing thioether groups into the system with respect to increasing the dielectric constant. The other dielectric characteristics remain commensurate with the previously obtained samples. It is also worth noting the fact of a decrease in dielectric parameters in opalescent samples having a high initial MS and PEOS content (Table 1, #18, 19), which, apparently, is a consequence of the reduced compatibility of components in the final material.

The use of modified rubber, in this case, determines the presence of high elasticity of the resulting compositions, even with the high filling of MS and PEOS. This effect is due to the possibility of multiplying the rubber chain due to its homocondensation under the catalytic action of the metal atom and residual amino groups. The elongation at the moment of rupture varies within ε~70–100%, depending on the silica filling degree. All the materials obtained were characterized by the absence of a neck formation when stretched, which indicates a high uniformity of the material. At the same time, the tensile strength values turned out to be quite low even with a high degree of silica filling (σ~0.4–2.9 MPa), inferior to those for compositions cured with phenyl silyl derivatives of Zr-siloxanes.

### 3.2. Compositions Cured with Ethoxy Silyl Derivatives of Sulfur-Containing Zr-Siloxanes

Based on the data obtained during the PDMS rubber curing with methoxy silyl derivatives ZrS1-3(4-0), a number of altered functionality MSs were synthesized, namely ethoxy silyl derivatives differing both in the metal atom shielding degree by siloxy substituents and in the configuration of the siloxy substituent.

For this, silane 4, which eventually contains half of the ethoxy silyl groups, was synthesized the same way as silane 3 by using vinyltriethoxysilane. Rebrov’s salt SiONa4 was obtained similarly to SiONa3. At the same time, ^1^H NMR spectroscopy showed that the methyl silyl group mainly reacts with alkali: the integral intensity of ethoxy groups on the SiONa4 spectrum practically does not change relative to silane 4 even after diffusion filtration (see Figure S1b in Supplementary Materials). This fact is also confirmed by the 100% yield of salt relative to the substitution product of one methoxy group. 

**Figure 6:**
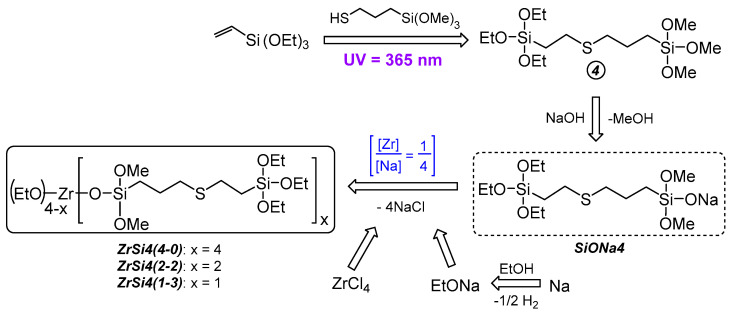
Scheme of ZrS4(4-0), ZrS4(2-2) and ZrS4(1-3) synthesis.

The use of SiONa4 made it possible to obtain MSs with a predominant content of ethoxy silyl groups. ZrS4(4-0) was an ethoxy silyl analog of ZrS3(4-0), and partially siloxy substituted ZrS4(2-2), and ZrS4(1-3) were obtained using silane 4, and sodium ethylates together to obtain the mole ratio of substituents at the metal atom ZrOSi/ZrOEt = 1:1 and 1:3 respectively (Figure 6). Thus, the ZrS4(4-0)→ZrS4(2-2)→ZrS4(1-3) row allowed us to further evaluate both the curing activity of MSs and the resulting compositions properties depending on the degree of metal atom shielding similarly to a number of phenyl silyl derivatives Zr-Ph(4-0)→Zr-Ph(2-2)→Zr-Ph(1-3) [34]. It is worth noting the greater stability over time of ZrS4(4-0) and its greater solubility in toluene, which is characterized by a greater reaction product yield compared to its analog ZrS3(4-0) due to the presence of a larger proportion of the organic part.

**Figure 7:**
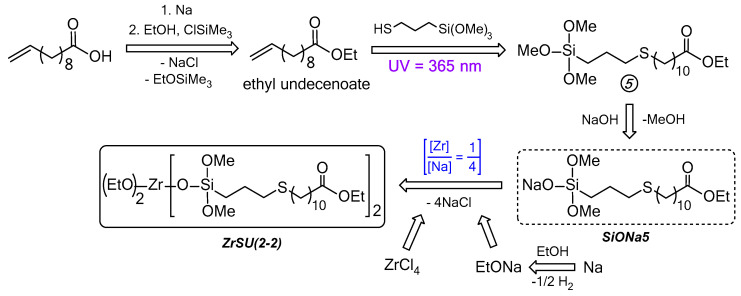
Scheme of ZrSU(2-2) synthesis.

Also, similarly to ZrS4(2-2), a partially siloxy-substituted ZrSU(2-2) was obtained, differing in the siloxy substituent structure. For this purpose, silane 5 was obtained by hydrothiolation of ethyl undecenoate with 3-mercaptopropyltrimethoxysilane under UV irradiation, followed by the interaction of the product with NaOH to obtain the Rebrov’s salt SiONa5 (Figure 7). This made it possible to evaluate the effect of a volumetric substituent on the MS activity, as well as the presence of ester groups, a long alkyl spacer in combination with a thioether fragment, on the properties of the cured material.

Synthetic relationship in this case can be presented as silane 4→SiONa4→ZrS4(4-0)/ZrS4(2-2)/ZrS4(1-3) and silane 5→SiONa5→ZrSU(4-0).

The elemental composition of obtained products is in good agreement with the calculated values. Nevertheless, the ^1^H NMR study showed some features. Thus, proton signals of ZrS4(4-0) fully correspond to the calculated proton content, which confirms the thesis that NaOH reacts only with the methoxy group in this case. However, the spectra of partially siloxy substituted products ZrS4(2-2) and ZrSU(2-2) contain signals of alkoxy groups (apparently, methoxy groups), partially shifted from the range of δ_H_ ~ 3.5–4.0 ppm into the region of the weak field δ_H_~1.25 ppm characteristic in this case for –OCH_2_CH_3_ protons. This fact is also confirmed by the ^1^H-^29^Si NMR HSQC spectrum for ZrS4(2-2), on which the correlation of the signal δ_Si_~−48.5 ppm with the signal δ_H_~1.20–1.40 ppm is also noticeable in this case. But it is not possible to distinguish clearly ethoxy groups at the silicon and metal atom in the spectrum. The presence of several signals in the ^29^Si NMR spectrum indicates the mixture of products, including partially hydrolyzed and oligomer ones (see Figure S2 in Supplementary Materials).

Table 2a–c presents data on the dielectric and mechanical properties of compositions cured with MSs ZrS4(4-0), ZrS4(2-2), and ZrSU(2-2). Data were obtained using ZrS4(1-3) as a hardener see Table S1 in Supplementary Materials. 

**Figure 8:**
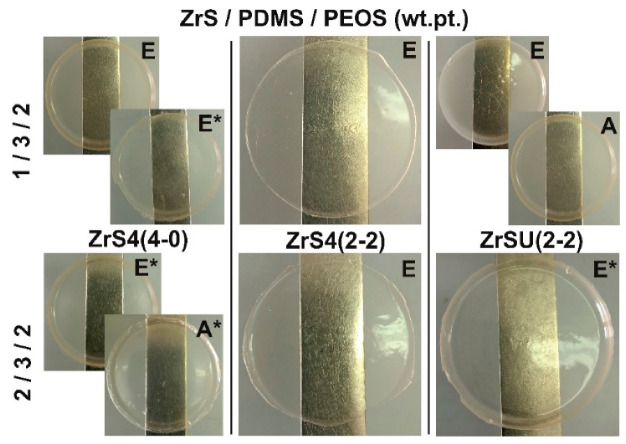
Compositions photos with the original ratio ZrS_X_/PDMS(A,E)/PEOS = 1/3/2 and 2/3/1 wt.pt.

As can be seen, the use of the ethoxy silyl analog of ZrS4(4-0) leads to compositions with similar dielectric characteristics. At the same time, poor quality of films is observed when using silanol-terminated rubber (Table 2a #5, 6). However, the final material shows a higher uniformity. Homogeneous elastomeric compositions were obtained using pre-blocked PDMS-A* and E* rubbers in the case of ZrS4(4-0) (Figure 8). There is a tendency to increase the dielectric constant in the low-frequency region with the increase in both the proportion of PEOS and MS in the initial composition. At the same time, it is possible to overcome the ε′ = 5 limits at f ≤ 10 Hz in some cases. Thus, with the initial composition of ZrS4(4-0)/PDMS-E*/PEOS = 2/3/2 wt.pt. it was possible to achieve the ε′ = 5 value even at f = 100 Hz (Table 2a #7). The use of lower molecular weight rubber (PDMS-A*) leads to a certain decrease in the values of ε′ in the f ≤ 10 Hz range, presumably due to a decrease in the proportion of inorganic mesh formation due to an increase in the rubber functional groups in the system. However, it still remains relatively high, with a positive effect on the elastic properties of the material even with the maximum filling of the silica component.

**Figure 9:**
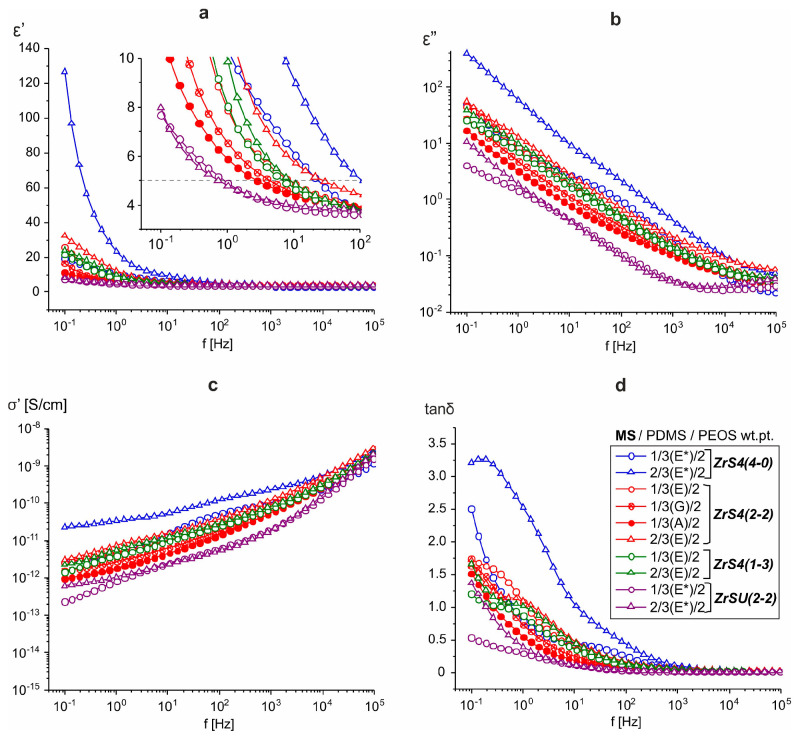
Frequency dependences of dielectric constant ε′ (**a**), dielectric losses ε″ (**b**), conductivity σ′ (**c**), and dielectric loss tangent tanδ (**d**) for three-component compositions ZrS4 or ZrSU/PDMS-E/PEOS at different initial ratios. The rubber brand is indicated in parentheses.

The use of non-fully siloxy-substituted derivatives ZrS4(2-2) and ZrS4(1-3) allows to get cured homogeneous elastomeric compositions using silanol terminated rubbers even with a high initial content of MS and PEOS (Figure 8). At the same time, there is also a tendency to increase the dielectric constant in the low-frequency region with an increase in both the initial proportion of PEOS and MS. However, there is a decrease in dielectric parameters in the ZrS4(4-0)→ZrS4(2-2)→ZrS4(1-3) row, which is most likely determined by a decrease in the number of thioester groups in the initial system (Figure 9). At the same time, the activity increases relative to the condensation reaction of all components of the initial mixture. In the case of using ZrS4(1-3), visually more homogeneous, but at the same time, more fragile compositions are obtained with an increase in the filling of MS and silica components. Such effect was observed earlier when using a similar series of phenyl derivatives of MSs Zr-Ph(4-0)→Zr-Ph(2-2)→Zr-Ph(1-3), explained by an increase in the probability of the ZrOx units’ formation in the cured system. In this case, it is possible to obtain cured compositions with a minimum content of MS component, but the effect of introducing sulfur will be small. In general, it is possible to achieve a significant increase of ε′ in the low-frequency region only in the case of PDMS-E rubber curing with ZrS4(2-2) with the maximum possible MS and silica components filling (Table 2b #10, 12, 13, 16). At the same time, there is a noticeable decrease in dielectric parameters with a decrease in the rubber chain length, which can be explained by an increase in the silanol groups proportion in the initial system and, as a consequence, a decrease in the three-dimensional silica mesh proportion formed from MS and PEOS. But in all cases, one can notice a significant effect of the introduction of sulfur atoms into the structure of the formed material on increasing the dielectric parameters, comparing the obtained ones in the case of phenyl silyl derivatives Zr-Ph(2-2) and Zr-Ph(1-3) using (Table 2b #19–22). A characteristic feature of non-fully siloxy substituted ZrS4(2-2) and ZrS4(1-3) using was their high activity, manifested in an increase in the viscosity of the initial system when kneading it even under conditions of strong dilution (noted in Table 2b and Table S1). This behavior was observed only when silanol rubbers using and, presumably, under conditions of a commensurate silanol and ethoxy silyl groups ratio in the system. It has a different character: from a slight increase in viscosity to an instantaneous transition to a gel-like state with the addition of MS. Moreover, the initial gel-like state eventually turned into a fluid high-viscosity state, which eventually allowed the system to be poured onto the substrate and form the films. 

The use of ZrSU(2-2) made it possible to obtain a homogeneous elastomeric material, mainly in the case of curing pre-blocked rubbers. This behavior can be explained by the difference in the kinetics of the PDMS and MS-PEOS network formation due to the reduced metal atom catalytic activity by shielding with bulk undecyl substituents. Good compositions were obtained with low molecular weight silanol terminated PDMS-A and G rubbers, i.e., the systems with a large initial content of silanol groups (Figure 9). The dielectric parameters, in this case, are noticeably lower than in the case of ZrS4(2-2), even at high MS and PEOS filling, which may indicate a low dielectric activity of the substituent at the silicon atom (Table 2c).

**Figure 10:**
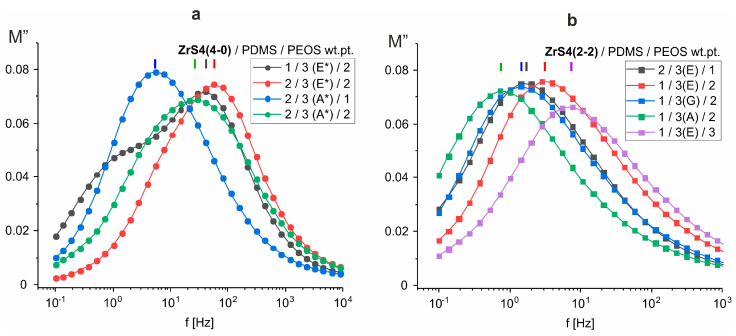
Frequency dependences of the imaginary part of electric modulus M″ for systems cured with ZrS4(4-0) (**a**) and ZrS4(2-2) (**b**).

According to the dielectric properties of the films, all the materials obtained are typical dielectrics. As in the case of using other zirconium siloxane derivatives, the dielectric constant at frequencies above 10^3^ Hz weakly depends on the material composition, ε′~3–4 (Figure 5a and Figure 9a). In this frequency range ε′ is determined by the presence of small polar groups (silanol groups) in the material. The concentration of such groups, apparently, is small and is practically independent of the composition, resulting in constant high-frequency permittivity. A sharp increase in ε′ at f < 10^2^ Hz is also accompanied by an increase in dielectric losses (Figure 5b,d and Figure 9b,d). At the same time, the frequency dependence of conductivity σ′ at low frequencies does not reach a plateau (Figure 5c and Figure 9c), i.e., there is no bulk dc conductivity (the slope of the frequency dependence σ′ ≈ 1) [39]. At the same time, both real ε′ and imaginary ε″ parts of the dielectric constant exhibit approximately linear behavior in log-log coordinates with the same slope of ~0.6. In the absence of bulk conduction, and also taking into account that the conductivity should not affect ε′, this means that the dominant process at low frequencies in these systems is the transfer of charge carriers in some limited, isolated regions [40,41], which may be residual silanol groups. This behavior is described by the so-called “quasi-DC” (QDC) model [40], when there are dipoles in the system, in which charges are weakly connected to each other so that they can separate and move as quasi-free ions at low frequencies. Maxima has observed the frequency dependences of the imaginary electrical modulus M″ of SiO_2_-containing systems (Figure 10). Their displacement towards the high-frequency region correlates with an increase in the dielectric constant of the material, i.e., with an increase in the SiO_2_ content [42]. In the QDC model, the appearance of an M″ maximum on the frequency dependencies is precisely associated with the transfer of charges in some isolated regions, while the position of the M″ maximum corresponds to the beginning of the ε′ increase (in logarithmic coordinates). At the same time, these regions should be interconnected, which in this case can be ensured by the formation of a continuous, evenly distributed MS-SiO_2_ network, and the binding effect is enhanced by the presence of thioester fragments.

**Figure 11:**
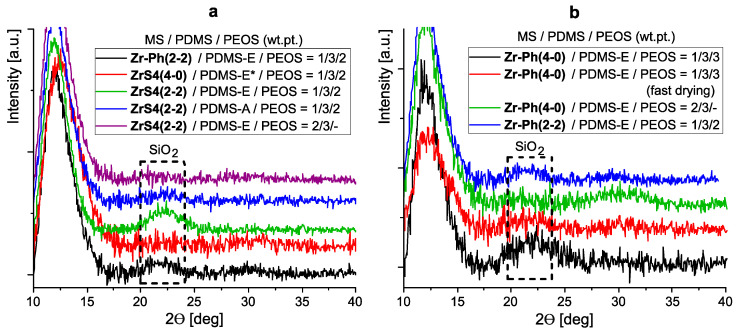
X-ray data for ~100 µm films formed using sulfur-containing ZrS4(4-0) and ZrS4(2-2) and previously obtained using Zr-Ph(4-0) and Zr-Ph(2-2).

The presence of separate silica domains is confirmed by X-ray scattering data. Diffractograms of some samples (Figure 11) obtained in the form of ~100 µm films in the region of Ɵ~21–22° show a broadened reflex corresponding to the presence of an amorphous silica phase [43]. Moreover, as thioether-containing compositions show, the reflex is characteristic only for three-component systems containing PEOS as a SiO_2_ precursor and silanol-terminated rubber curing (Figure 11a). Thus, the reflex is practically absent in the case of a pre-blocked PDMS-E*, i.e., completely ethoxy silyl initial system. Also, its intensity decreases when using lower molecular weight rubber (PDMS-A), i.e., with an increase in the Si-OH system functionality and, as a consequence, greater binding of the initial PEOS functional groups with rubber and a decrease in the size of formed SiO_2_ domains. Similar diffractograms are observed for systems cured with previously synthesized phenyl-containing MSs (Figure 11b). At the same time, the reflex corresponding to silica is observed even in the case of accelerated drying of the initial system.

The use of pre-blocked rubber determines the presence of increased elasticity of the resulting compositions similar to the ZrS3(4-0) used. However, its values are noticeably lower than those for ZrS3(4-0) (ε~4–74%) in the case of PDMS-E*, and the lower, the higher the degree of MS-SiO_2_ filling. At the same time, elasticity decreases with a decrease in the length of the rubber chain. However, even in the case of PDMS-A* curing, an elastomeric material can be obtained with the very high introduction of a silica component with elastic deformations proportion predominant (Table 2a #16). On the contrary, the tensile strength increases with the use of ethoxy-derived MS and can reach σ~6 MPa values, which is comparable with those for compositions cured with phenyl silyl derivatives of zirconium siloxanes.

Compositions cured with ZrS4(2-2) and ZrS4(1-3) are characterized by previously established correlations when using phenyl silyl derivatives of zirconium siloxanes [34]. In general, compositions that do not contain SiO_2_ components are the most elastic. The silica component presence, in combination with the MS network, contributes to the material hardening with a decrease in its elasticity. This is more pronounced for the ZrS4(1-3), which may be due to greater activity regarding the formation of ZrOx domains in the system. The maximum elongation values (ε up to 150–300%) were observed during the PDMS-E rubber curing with ZrS4(2-2) in the case of a reduced proportion of initial MS component (Table 2b #1–3, 6). However, such systems are generally characterized by a small proportion of elastic deformations with a predominance of rupture during neck propagation. The presence of a high functionality of the organic substituent at the silicon atom (in comparison with phenyl silyl derivatives of MSs) determines the formation of a denser inorganic network and, as a consequence, reduced elasticity. At the same time, a characteristic feature here is a decrease in elasticity and an increase in strength with a decrease in the rubber chain length, which is a consequence of an increase in the network density due to an increase in the functionality of the initial system. The opposite effect was observed in the case of phenyl silyl MSs. The strength values of the compositions vary in the range σ~2.3–9.0 MPa. Such an effect in the future can be minimized by using pre-blocked rubbers.

**Figure 12:**
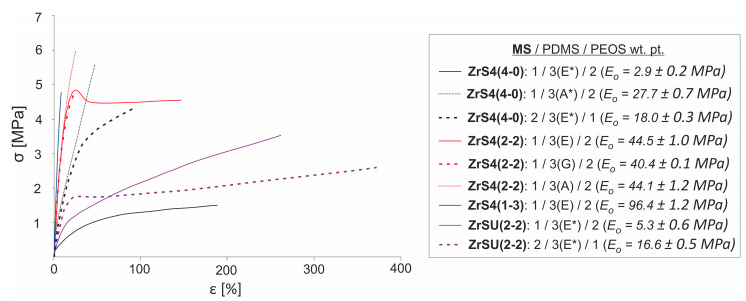
Examples of stretch curves for compositions differing in MS and its initial content, as well as in the rubber (with numerical values of the elasticity modulus E_o_).

Compositions cured with ZrSU(2-2) are in an intermediate position in terms of mechanical characteristics. On the one hand, they have greater elasticity compared to those cured by ZrS4(2-2), even in the case of silanol-terminated rubber using, which is determined by the lower condensation activity of the organic fragment and the formation of a less dense three-dimensional grid. On the other hand, there is a tendency to have a large proportion of irreversible deformations when using pre-blocked PDMS-E*, unlike compositions cured with ZrS4(4-0). Accordingly, their strength and elongation values fit into the ranges of values for the compositions considered above. Characteristic stretching curves illustrating the described correlations are shown in Figure 12. The given values of the elastic modulus (E_o_) correlate well with the filling degree, and silica component filling makes a greater contribution to hardening. At the same time, the use of pre-blocked rubber leads to lower values of the elastic modulus. However, in this case, rubber chain length adjusting can be a tool for increasing it.

**Figure 13:**
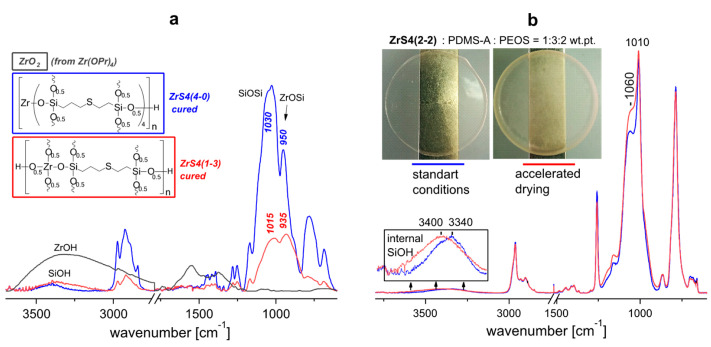
IR spectra: (**a**)—condensed ZrS4(4-0) and ZrS4(1-3) in comparison with ZrO_2_; (**b**)—films with the initial of ZrS4(2-2)/PDMS-A/PEOS = 1/3/2 wt.pt. composition at different drying rates.

The previously discovered effect of the film drying rate on the mechanical properties was tested in the compositions. Since it is most justified in the presence of heterofunctional condensation, i.e., in the case of curing of silanol-terminated rubber, the effect was considered for compositions cured with ZrS4(2-2) (Table 2b #9, 13, 17). If the total initial reagent concentration in the solution was 10–15% for standard curing, then it was ~30% for accelerated drying. The heterofunctional condensation (i.e., PDMS network formation) processes have enough time and opportunity to convert more fully in the case of faster drying than all the processes of the MS-SiO_2_ network formation and its uniform distribution. Indeed, opalescence is observed almost in all cases (Figure 13b), which indicates the heterogeneity of the components’ distribution in material, and the composition mechanics also change: the strength and the proportion of elastic deformations increase. However, there is a noticeable decrease in dielectric characteristics. It is worth noting that in the case of phenyl silyl derivatives using faster-drying films tended to decrease the strength of the material. There is redistribution of the band’s intensity in the 900–1100 cm^−1^ region on the IR spectra of films cured by different methods: the intensity of the band characteristic of the Si-O-Si bond is reduced, while the intensity of the band {Si-O-Si + Zr-O-Si} bond is kept (Figure 13a). This fact also speaks in favor of a slightly reduced conversion of the homofunctional condensation of the alkoxy groups of MS and PEOS in the thickness of the material with the formation of a Si-O-Si bond. Observed residual OH-groups (SiOH and ZrOH) inside the material (they are not observed on the film surface) provided high dielectric constant, and the maxima peak are somewhat shifted relative to each other depending on the drying rate (Figure 13b).

**Figure 14:**
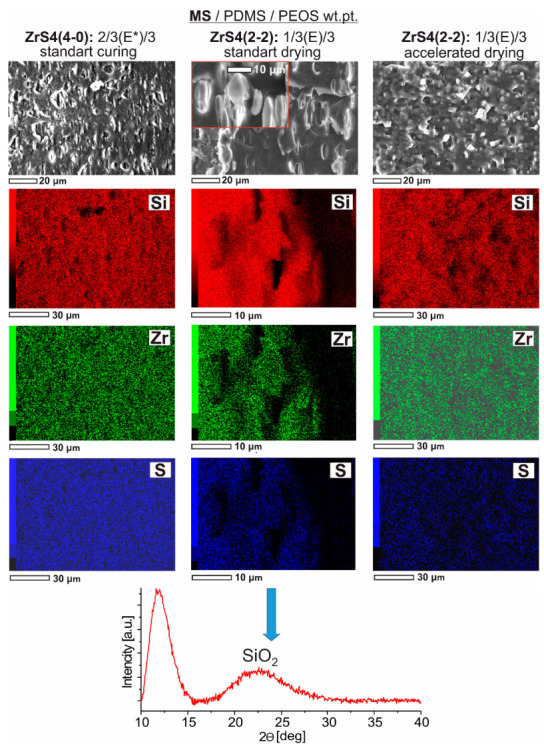
SEM data and element maps for some compositions were obtained using ZrS4(4-0) and ZrS4(2-2).

The presence of an increased number of alkoxy groups compared to previously studied compositions presumably determines their porosity caused by alcohol presence formed after condensation and its departure through the film thickness during the heating process (Figure 14). At the same time, according to the element maps data, we have a uniform key elements distribution in the volume of material, which indicates a high uniformity and a high degree of mutual penetration of MS, silica, and PDMS networks. The drying rate effect on the forming material morphology is noticeable. So, for example, for the ZrS4(2-2)/PDMS-E/PEOS = 1/3/3 wt.pt. initial composition, it can be seen that under standard conditions, some anisotropic formations can form in the material thickness, which is absent in the case of accelerated drying. This effect is also reproduced when using PDMS-A* rubber. The fact that we have foreign particles, not a pore, is indicated by a number of factors, such as an oblong shape, alignment in one direction, as well as elemental analysis data of these areas, showing negligible zirconium and sulfur content on their surface. It is possible to assume the silica nature of such inclusions. At the same time, the diffractogram of such composition film has a more pronounced broadened reflex corresponding to the silica phase, compared with the previously given compositions (Figure 11), which also confirms this fact.

### 3.3. Compositions Cured with a Mixture of Zi-Siloxanes

It was also interesting to investigate the effect of MS combinations on the final material properties (Table 3). The most successful earlier investigated compositions MS/PDMS(G,E)/PEOS = 1(0.5)/3/2(3) wt.pt. in terms of dielectric and mechanical properties were taken as the basis (where MS is phenyl- or vinyl-containing Zr-siloxane) and a part of the initial MS was replaced with thioether-containing MS.

This is important, in particular, for ZrS4(4-0) because, in this case, it is possible to avoid the need for pre-blocked rubber when using a “more active” hardener relative to silanol-terminated PDMS. Thus, the addition of a part of full siloxy substituted Zr-Ph(4-0) does not lead to the material with sufficient elastomeric properties, and the use of more active Zr-Ph(2-2) and Zr-Ph(1-3) containing Zr-OEt groups, on the contrary, results in an elastomeric material. Moreover, in the case of Zr-Ph(1-3), it is possible to increase the proportion of ZrS4(4-0) (up to 0.75) due to the higher Zr-OEt groups content in the system, thereby affecting the kinetics of networks formation (Table 3 #7). However, this approach does not work when ZrS4(1-3) is added as a co-hardener. Comparing the characteristics of materials obtained with those obtained with only Zr-Ph derivatives, it can be concluded that replacing part of the initial amount of MS with sulfur-containing ZrS4(4-0) significantly increases the dielectric parameters in the f < 10 Hz range. 

The combination of “more active” (containing Zr-OEt groups) MSs makes it possible to obtain homogeneous elastomeric materials in almost all of the above cases. There is also a noticeable increase in dielectric parameters with the addition of thioester-containing ZrS4(2-2) and ZrSU(2-2). Moreover, the increase of ε′ in the frequency range f < 10^2^ Hz is most pronounced in the case of their combination with vinyl-containing Zr-Vin(2-2) (Table 3 #13–16). This can presumably be explained by the presence of some additional interactions of a non-covalent nature due to the presence of both unsaturated bonds and electron-donor thioester groups in the system. At the same time, the use of a combination of thioester MS practically does not affect the dielectric properties of the material, in particular, relative to the initial content of ZrS4(2-2) (Table 3 #28, 29).

**Figure 15:**
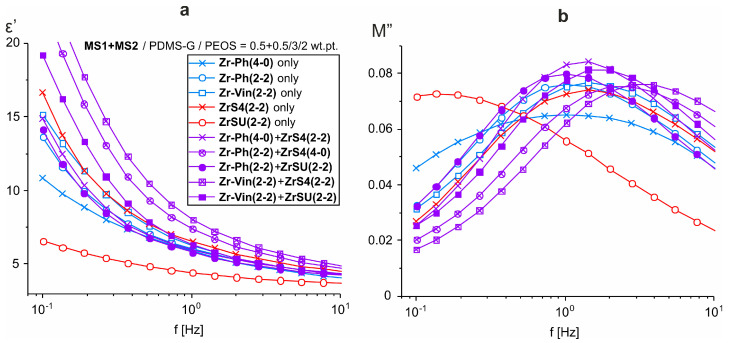
Frequency dependence comparison of dielectric constant (**a**) and imaginary modulus (**b**) for compositions cured with various MSs and their mixtures.

In general, the same dependences of dielectric and mechanical properties on the initial PEOS content and rubber molecular weight remain as in previous cases when MS mixtures are used. An illustration of the effect of the sulfur atom’s introduction is clearly shown in the frequency dependences of the dielectric constant and imaginary modulus (Figure 15).

The combination of different MS is associated with a change in mechanical properties of compositions compared to those that do not contain a thioester MS network. Against the background of a slight decrease in tensile strength, the elasticity of the material also significantly decreases, presumably due to an increase in the cross-linking density. In addition, in most cases, the materials obtained are characterized by the formation of the neck during stretching. However, as before, the presence of a neck can be affected by changing the drying rate in the air (see Figure S3 in Supplementary Materials).

## 4. Conclusions

In this work, a number of thioether-containing zirconium siloxanes were synthesized, differing in their composition and metal atom shielding degree with a siloxy substituent. A wide range of highly filled compositions based on PDMS rubber with different chain lengths, cured with thioester-containing metallosiloxanes, has been obtained. The dielectric and mechanical properties of the obtained compositions were investigated. Data obtained made it possible to evaluate the activity of sulfur-containing zirconium siloxanes as hardeners of silicone compositions, as well as the effect of the sulfur atom presence on materials’ dielectric properties compared to previously studied. Varying the filling degree within a fairly wide range allowed us to assess the effect of the ratio and type of the initial components on the final material properties. It is shown that the resulting systems also behave like typical dielectrics. At the same time, the introduction of thioether groups in combination with an in situ formed silica component with the zirconium siloxane network formation can provide increased dielectric constant (up to ~10–30) and conductivity (up to ~0.1–12 S/cm) not only in comparison with industrial silicones and pure PDMS but also with previously obtained sulfur-free similar compositions in the <10^2^ Hz frequency range. As before, the dielectric parameters increase is directly determined by the silica component proportion in the cured material. The character of frequency dependences of dielectric parameters determines the increase of their values due to the transfer of charge carriers in the inorganic network clusters. The introduction of a proportion of sulfur-containing MS, along with varying the functionality and rubber chain length, can be an effective tool for changing the dielectric and mechanical material parameters (tensile strength 0.5–7 MPa, elastic deformation 2–300%), which determines the prospects for the use of such cured systems as dielectric elastomers for various purposes. In addition, due to the effectiveness of such an approach, a number of functional metallosiloxanes as hardeners of silicone compositions will be expanded in the future, including ones containing other heteroatoms and functional groups. Separately, the production of new thioether containing Rebrov’s salts allows the future to create other functional polysiloxanes. This approach will allow the use of functional alkoxysilanes as additives to impart special properties to silicone elastomeric materials.

## Figures and Tables

**Table 1:** Compositions characteristics obtained using ZrS1(4-0), ZrS2(4-0) and ZrS3(4-0).

No.	Initial Ratio MS/PDMS-E/PEOS wt.pt.		ε′	ε″	σ′·10^11^ S/cm	tanδ	$\frac{\sigma \pm \Delta \sigma }{\varepsilon \pm \Delta \varepsilon }$ MPa/%	Characterization
			f = 0.1/1/10/100 Hz					
1	ZrS1(4-0)	1/3/-	-	-	-	-	-	Transparent, waxy
2		1/3/1	-	-	-	-	-	
3		1/3 * /-	-	-	-	-	-	White, irreversibly deformed
4		2/3 * /1	10.6/6.1/4.4/4.1	7.2/2.3/0.7/0.2	0.04/0.13/0.39/0.97	0.68/0.38/0.16/0.05	1.2 ± 0.1 70 ± 11	Opalescent, heterogeneous
5		1/3 * /2	4.7/4.4/3.8/3.4	0.1/0.3/0.4/0.2	0.001/0.02/0.22/0.96	0.02/0.07/0.11/0.06	1.2 ± 0.1 161 ± 10	White, heterogeneous
6	ZrS2(4-0)	1/3/-	-	-	-	-	-	Not cured
7		1/3 * /-	2.5/2.3/2.3/2.3	0.2/0.1/0.02/0.01	0.001/0.004/0.01/0.06	0.08/0.03/0.01/0.004	0.4 ± 0.03 100 ± 15	Transparent, yellowish, homogeneous
8		1/3 * /1	5.0/4.3/3.7/3.4	0.8/0.4/0.3/0.2	0.005/0.02/0.19/0.93	0.16/0.09/0.08/0.06	1.6 ± 0.2 261 ± 38	
9		2/3 * /1	16.4/7.2/4.5/3.8	28.0/5.5/1.32/0.4	0.16/0.31/0.79/2.12	1.70/0.76/0.29/0.11	3.7 ± 0.1 205 ± 5	
10		1/3 * /2	20.7/10.4/5.9/4.2	24.6/6.4/2.2/0.9	0.14/0.36/1.30/5.22	1.19/0.61/0.37/0.21	2.1 ± 0.1 264 ± 16	
11		2/3 * /2	34.1/17.0/6.7/4.6	222.7/30.2/5.5/1.3	1.24/1.71/3.28/7.07	6.53/1.78/0.82/0.28	3.3 ± 0.1 89 ± 19	
12	ZrS3(4-0)	1/3/-	-	-	-	-	-	Heterogeneous, waxy
13		1/3 * /-	3.5/3.5/3.3/3.1	0.04/0.06/0.1/0.1	0.0002/0.003/0.07/0.80	0.01/0.02/0.03/0.03	0.6 ± 0.1 121 ± 18	Transparent, yellowish, homogeneous
14		1/3 * /1	4.6/3.9/3.6/3.2	1.0/0.4/0.3/0.2	0.01/0.02/0.16/0.11	0.22/0.10/0.08/0.06	1.8 ± 0.3 189 ± 45	
15		2/3 * /1	10.5/8.9/7.3/5.7	2.7/1.2/1.1/1.1	0.02/0.07/0.65/6.19	0.26/0.13/0.15/0.19	2.2 ± 0.4 165 ± 29	
16		1/3 * /2	11.7/6.0/4.7/3.9	9.6/2.3/0.8/0.5	0.05/0.13/0.45/2.49	0.82/0.38/0.17/0.13	2.9 ± 0.3 325 ± 41	
17		2/3 * /2	40.4/17.3/7.7/4.8	223.0/29.7/5.8/1.6	1.24/1.69/3.48/9.03	5.5/1.72/0.75/0.33	2.7 ± 0.2 158 ± 21	
18		2/3 * /3	18.6/13.4/10.5/7.6	16.1/3.6/2.1/2.1	0.09/0.20/1.25/1.19	0.87/0.27/0.2/0.28	2.2 ± 0.2 266 ± 23	Opalescent, yellowish, homogeneous
19		2/3 * /3 ~100 μm	8.8/7.0/6.1/4.9	5.4/1.2/0.7/1.0	0.03/0.07/0.43/5.92	0.61/0.17/0.11/0.20	2.9 ± 0.1 310 ± 40	
20	Zr-Ph(4-0)	1/3/2	10.3/7.1/4.5/3.8	2.9/2.0/1.2/0.3	0.02/0.11/0.69/1.68	0.28/0.28/0.25/0.08	5.6 ± 0.3 14 ± 2	Transparent, yellowish, homogeneous
21		1/3 * /2	8.6/5.1/3.8/3.4	6.5/2.0/0.6/0.2	0.04/0.11/0.33/0.87	0.76/0.38/0.15/0.05	5.2 ± 0.5 347 ± 32	
22	Ecoflex ^TM^		3.4/3.2/3.1/3.1	0.2/0.1/0.05/0.03	0.001/0.004/0.03/0.17	0.90/0.37/0.12/0.04	-	White

* PDMS-E pre-blocked with 3-aminopropyltriethoxysilane; previously obtained data are indicated in gray; ε′—dielectric constant, ε″—dielectric losses, σ′—conductivity, tanδ—dielectric loss tangent, σ/ε—tensile strength/elongation at the moment of film rupture.

**Table 2:** Compositions characteristics were obtained using ZrS4(4-0).

(a) Compositions Characteristics Obtained Using ZrS4(4-0)								
No.	Initial Ratio ZrS4(4-0)/PDMS/PEOS wt.pt.		ε′	ε″	σ′·10^11^ S/cm	tanδ	$\frac{\sigma \pm \Delta \sigma }{\varepsilon \pm \Delta \varepsilon }$ MPa/%	Characterization
			f = 0.1/1/10/100 Hz					
1	2/3(E *)/1		14.3/7.0/4.5/3.6	16.1/3.7/1.3/0.4	0.12/0.40/2.22/11.47	1.12/0.53/0.28/0.90	4.4 ± 0.1 92 ± 10	Transparent, yellowish, homogeneous
2	2/3(A *)/1		26.1/8.7/5.1/4.0	94.3/13.3/2.4/0.6	0.52/0.76/1.43/3.29	3.61/1.53/0.47/0.15	4.5 ± 0.3 65 ± 5	
3	1/3(E *)/2		19.3/10.3/6.0/3.7	48.2/8.1/2.7/0.9	0.27/0.46/1.59/5.24	2.50/0.78/0.44/0.24	1.6 ± 0.1 174 ± 29	Transparent, yellowish, homogeneous, light plasticity effect
4	1/3(A *)/2		5.6/3.8/3.4/3.3	4.1/0.9/0.2/0.1	0.02/0.05/0.12/0.31	0.74/0.23/0.06/0.02	5.5 ± 0.2 42 ± 3	Transparent, yellowish, homogeneous
5	1/3(E)/2		-	-	-	-	-	Opalescent, yellowish, homogeneous, brittle
6	1/3(A)/2		-	-	-	-	-	
7	2/3(E *)/2		126.6/23.4/9.0/5.0	405.1/58.9/9.1/2.2	2.25/3.34/5.42/12.06	3.20/2.52/1.01/0.42	2.5 ± 0.3 4 ± 1	
8	2/3(A *)/2		50.8/16.1/7.0/4.4	109.8/22.0/4.5/1.3	0.61/1.25/2.71/7.24	2.16/1/37/0.65/0.28	5.7 ± 0.8 71 ± 18	Transparent, yellowish, homogeneous
9	1/3(E *)/3		8.4/6.9/4.5/3.7	1.2/1.3/1.2/0.3	0.04/0.34/2.18/11.53	0.14/0.19/0.26/0.08	2.5 ± 0.2 295 ± 34	
10	1/3(A *)/3		7.8/4.2/3.5/3.4	8.0/1.6/0.3/0.1	0.05/0.09/0.19/0.43	1.03/0.37/0.09/0.02	6.0 ± 0.4 47 ± 4	
11	2/3(E *)/3		16.7/9.3/5.0/4.0	17.6/4.9/1.8/0.4	0.13/0.55/2.63/17.40	1.05/0.53/0.36/0.10	4.2 ± 0.1 74 ± 7	
12	2/3(A *)/3		37.5/16.2/7.4/4.5	153.5/22.0/5.0/1.4	0.85/1.25/2.98/8.07	4.09/1.36/0.67/0.32	6.1 ± 0.3 83 ± 13	
13 ^b^	2/3(A *)/3		34.3/13.7/6.4/4.3	88.4/16.1/3.7/0.9	0.49/0.91/2.23/5.22	2.57/1.17/0.59/0.21	4.5 ± 0.3 57 ± 3	
14	2/3(A *)/5		27.2/12.7/7.1/4.4	51.4/9.9/3.3/1.1	0.29/0.56/1.95/6.02	1.89/0.78/0.46/0.25	5.7 ± 0.3 22 ± 3	Transparent, yellowish, homogeneous, plasticity effect
**(b) Compositions Characteristics Obtained Using ZrS4(2-2).**								
**No.**	**Initial ratio** **ZrS4(2-2)/PDMS/PEOS wt.pt.**		**ε′**	**ε″**	**σ′·10^11^ S/cm**	**tanδ**	$\frac{\sigma \pm \Delta \sigma }{\varepsilon \pm \Delta \varepsilon }$ **MPa/%**	**Characterization**
			**f = 0.1/1/10/100 Hz**					
1 ^a^	1/3(E)/1		13.5/6.2/4.3/3.6	22.0/4.0/1.0/0.3	0.12/0.23/0.60/1.70	1.63/0.64/0.23/0.08	4.1 ± 0.2 ^d^ 322 ± 53	Transparent, yellowish, homogeneous
2 ^a^	0.5/3(E)/2		13.7/6.5/4.3/3.6	16.0/3.7/1.1/0.4	0.09/0.21/0.63/1.98	1.17/0.57/0.24/0.09	2.4 ± 0.2 249 ± 61	
3	0.5/3(G)/2		11.6/5.4/4.1/3.6	17.7/3.1/0.7/0.2	0.10/0.17/0.43/1.20	1.53/0.57/0.18/0.06	3.2 ± 0.2 19 ± 4	Transparent, yellowish, homogeneous, brittle
4	2/3(E)/1		16.8/6.6/4.4/3.8	22.7/4.9/1.1/0.3	0.13/0.28/0.66/1.74	1.35/0.74/0.25/0.08	9.0 ± 0.5 23 ± 5	Transparent, yellowish, homogeneous
5	2/3(G)/1		13.0/5.7/4.2/3.7	17.8/3.4/0.8/0.3	0.10/0.19/0.48/1.31	1.37/0.59/0.19/0.07	5.2 ± 0.2 6 ± 1	
6	1/3(E)/2		26.1/7.9/4.8/3.9	45.1/8.4/1.7/0.5	0.25/0.48/1.02/2.78	1.73/1.07/0.35/0.12	4.6 ± 0.1 ^d^ 166 ± 51	
7 ^a^	1/3(G)/2		16.6/6.5/4.5/3.8	27.0/4.7/1.1/0.3	0.15/0.27/0.65/1.85	1.62/0.72/0.24/0.08	4.8 ± 0.1 28 ± 2	
8 ^a^	1/3(A)/2		11.2/5.9/4.4/3.9	16.9/3.2/0.8/0.3	0.09/0.18/0.46/1.42	1.51/0.54/0.18/0.06	6.0 ± 0.4 26 ± 3	
9 ^a b^	1/3(A)/2		9.4/5.0/4.0/3.7	9.2/2.0/0.5/0.2	0.05/0.11/0.30/0.88	0.98/0.40/0.12/0.04	6.6 ± 0.6 14 ± 2	Opalescent, yellowish, homogeneous
10 ^a^	0.5/3(E)/3		31.1/9.7/5.4/4.2	58.2/11.6/2.2/0.6	0.32/0.66/1.34/3.52	1.87/1.19/0.42/0.15	2.7 ± 0.03 8 ± 1	Transparent, yellowish, homogeneous, light plasticity effect
11	0.5/3(G)/3		15.0/5.8/4.1/3.6	32.0/4.9/1.0/0.3	0.18/0.28/0.60/1.54	2.14/0.85/0.25/0.08	3.8 ± 0.2 12 ± 6	Transparent, yellowish, homogeneous, brittle
12 ^a^	2/3(E)/2		32.4/12.0/5.7/4.4	55.5/13.0/2.6/0.7	0.31/0.74/1.57/3.87	1.71/1.08/0.46/0.16	5.0 ± 1.0 4 ± 1	Transparent, yellowish, homogeneous
13 ^a b^	2/3(E)/2		29.6/11.8/5.6/4.2	36.9/10.8/2.5/0.7	0.21/0.61/1.48/3.86	1.25/0.92/0.44/0.16	6.2 ± 1.0 7 ± 1	Opalescent, yellowish, homogeneous, brittle
14	2/3(G)/2		26.7/7.9/5.0/4.2	33.7/7.3/1.5/0.4	0.19/0.42/0.89/2.23	1.26/0.93/0.30/0.10	3.0 ± 0.6 2 ± 1	Transparent, yellowish, homogeneous, brittle
15	2/3(A *)/2		13.6/6.0/4.4/4.0	16.1/3.3/0.8/0.2	0.09/0.19/0.46/1.29	1.18/0.55/0.18/0.06	3.4 ± 0.2 3 ± 1	
16	1/3(E)/3		37.1/12.4/6.1/4.6	71.0/14.6/2.9/0.8	0.39/0.83/1.76/4.56	1.91/1.17/0.49/0.18	5.0 ± 0.1 ^c^ 13 ± 1	Transparent, yellowish, homogeneous
17 ^a b^	1/3(E)/3		53.3/18.3/9.2/6.0	132.5/21.1/5.1/1.8	0.74/1.20/3.03/9.92	2.49/1.15/0.55/0.30	2.4 ± 0.1 20 ± 3	Opalescent, yellowish, homogeneous
18 ^a^	1/3(G)/3		27.5/8.5/5.3/4.3	54.2/9.6/1.9/0.5	0.30/0.54/1.11/3.06	1.97/1.12/0.35/0.13	6.2 ± 0.1 18 ± 1	Transparent, yellowish, homogeneous
19 ^e^	Zr-Ph(2-2)	1/3(E)/2	14.4/5.8/4.1/3.6	18.2/3.7/0.8/0.2	0.10/0.21/0.50/1.31	1.27/0.64/0.21/0.07	6.5 ± 0.1 35 ± 3	Transparent, yellowish, homogeneous
20 ^e^		2/3(E)/2	12.8/5.6/4.0/3.6	13.3/3.1/0.7/0.2	0.074/0.18/0.44/1.19	1.05/0.56/0.18/0.06	8.3 ± 0.2 14 ± 1	
21 ^e^	Zr-Vin(2-2)	1/3(E)/3	40.3/10.0/5.4/4.3	82.4/14.9/2.5/0.6	0.46/0.85/1.52/3.45	2.05/1.49/0.47/0.14	7.6 ± 0.7 9 ± 1	
22 ^e^		2/3(E)/2	13.0/5.4/3.9/3.6	12.5/3.0/0.7/0.2	0.07/0.17/0.40/1.08	0.96/0.56/0.17/0.05	5.9 ± 0.6 7 ± 1	
**(c) Compositions Characteristics Obtained Using ZrSU(2-2).**								
**No.**	**Initial ratio** **ZrSU(2-2)/PDMS/PEOS wt.pt.**		**ε′**	**ε″**	**σ′·10^11^ S/cm**	**tanδ**	$\frac{\sigma \pm \Delta \sigma }{\varepsilon \pm \Delta \varepsilon }$ **MPa/%**	**Characterization**
			**f = 0.1/1/10/100 Hz**					
1	1/3(E)/1		-	-	-	-	-	Transparent, yellowish, homogeneous, plasticity effect
2	2/3(E *)/1		7.6/4.7/3.8/3.6	10.9/1.9/0.4/0.1	0.06/0.11/0.26/0.58	1.45/0.41/0.12/0.03	2.7 ± 0.2 ^d^ 374 ± 50	Transparent, yellowish, homogeneous
3	1/3(E)/2		-	-	-	-	-	Transparent, yellowish, heterogeneous morphology
4	1/3(G)/2		6.6/4.4/3.7/3.5	4.6/1.2/0.3/0.1	0.03/0.07/0.19/0.43	0.70/0.26/0.08/0.02	2.1 ± 0.1 15 ± 0.4	Transparent, yellowish, homogeneous
5	1/3(A)/2		6.7/4.5/3.8/3.7	6.6/1.3/0.3/0.1	0.04/0.07/0.18/0.42	0.99/0.30/0.08/0.02	2.7 ± 0.2 28 ± 4	
6	1/3(E *)/2		7.7/4.9/3.9/3.6	4.1/1.4/0.4/0.1	0.02/0.08/0.25/0.62	0.53/0.29/0.11/0.01	3.5 ± 0.4 278 ± 45	
7	2/3(E *)/2		8.0/4.8/4.0/3.8	10.9/1.9/0.4/0.9	0.06/0.11/0.23/0.53	1.36/0.39/0.10/0.02	4.1 ± 0.1 ^d^ 142 ± 52	
8	2/3(A *)/2		6.9/4.8/4.2/4.0	5.9/1.2/0.3/0.1	0.03/0.07/0.18/0.45	0.85/0.26/0.07/0.02	5.0 ± 0.1 62 ± 9	
9	1/3(E)/3		-	-	-	-	-	Transparent, yellowish, heterogeneous morphology
10	1/3(G)/3		-	-	-	-	-	

* PDMS pre-blocked with 3-aminopropyltriethoxysilane; (a) ε′—dielectric constant, ε″—dielectric losses, σ′—conductivity, tanδ—dielectric loss tangent, σ/ε—tensile strength/elongation at the moment of film rupture; ^b^ accelerated composite drying; The rubber brand is indicated in parentheses.; (b) ε′—dielectric constant, ε″—dielectric losses, σ′—conductivity, tanδ—dielectric loss tangent, σ/ε—tensile strength/elongation at the moment of film rupture; ^a^ increase in the mixture viscosity with the MS addition; ^b^ accelerated composite drying; ^c^ rupture during the neck formation; ^d^ rupture during the neck spreading; ^e^ previous study data obtained with Zr-Ph(2-2) and Zr-Vin(2-2) using; (c) ε′—dielectric constant, ε″—dielectric losses, σ′—conductivity, tanδ—dielectric loss tangent, σ/ε—tensile strength/elongation at the moment of film rupture; ^d^ rupture during the neck spreading; The rubber brand is indicated in parentheses.

**Table 3:** Compositions characteristics were obtained using a mixture of Zr-siloxanes.

No.	Initial Ratio MS1+MS2/PDMS/PEOS wt.pt.		ε′	ε″	σ′·10^11^ S/cm	tanδ	$\frac{\sigma \pm \Delta \sigma }{\varepsilon \pm \Delta \varepsilon }$ MPa/%	Characterization
			f = 0.1/1/10/100 Hz					
1	ZrS4(4-0) + Zr-Ph(2-2)	0.5+0.5/3(G)/2	22.8/7.4/4.7/3.9	34.5/6.8/1.4/0.4	0.19/0.38/0.85/2.22	1.52/0.92/0.31/0.10	6.5 ± 0.1 ^d^ 49 ± 7	Transparent, yellowish, homogeneous
2		0.25+0.25/3(G)/3	70.9/20.2/7.2/5.1	105.4/28.4/5.1/1.3	0.58/1.61/3.04/6.93	1.49/1.40/0.71/0.24	6.8 ± 0.4 12 ± 1	
3		0.25+0.25/3(E)/3	94.1/28.8/8.5/5.5	240.7/45.3/8.2/1.8	1.34/2.57/4.90/10.40	2.56/1.57/0.96/0.33	4.3 ± 0.2 ^d^ 202 ± 51	
4	ZrS4(4-0) + Zr-Vin(2-2)	0.25+0.25/3(E)/3	7.9/4.8/3.9/3.7	7.2/1.6/0.4/0.1	0.06/0.23/1.77/15.60	0.91/0.32/0.10/0.03	3.9 ± 0.1 ^d^ 24 ± 1	Transparent, yellowish, homogeneous
5		0.25+0.25/3(G)/3	6.1/4.4/3.9/3.8	4.5/0.9/0.2/0.1	0.04/0.20/16.21	0.73/0.21/0.05/0.02	7.7 ± 0.3 7 ± 1	
6		0.25+0.25/3(G)/3	6.1/4.6/3.9/3.7	1.9/0.8/0.3/0.1	0.03/0.21/1.71/15.45	0.31/0.18/0.07/0.02	3.5 ± 0.1 23 ± 2	Opalescent, yellowish, homogeneous
7	ZrS4(4-0) + Zr-Ph(1-3)	0.75+0.25/3(E)/2	35.4/13.8/5.9/3.9	184.9/26.0/4.5/1.2	1.03/1.47/2.72/6.66	5.23/1.88/0.77/0.31	3.9 ± 0.1 18 ± 3	Transparent, yellowish, homogeneous
8 ^a^	ZrS4(4-0) + ZrS4(2-2)	0.5+0.5/3(G)/2	18.2/7.3/4.4/3.6	21.1/4.9/1.4/0.4	0.12/0.28/0.82/2.03	1.16/0.67/0.31/0.10	3.1 ± 0.1 24 ± 2	Transparent, yellowish, homogeneous
9	ZrS4(2-2) + Zr-Ph(4-0)	0.5+0.5/3(G)/2	14.9/5.9/4.3/3.8	33.0/5.0/1.0/0.3	0.18/0.28/0.60/1.52	2.22/0.84/0.23/0.07	4.9 ± 0.1 ^d^ 81 ± 6	Transparent, yellowish, homogeneous
10		0.25+0.25/3(G)/3	16.7/6.7/4.6/3.9	23.7/4.7/1.1/0.4	0.13/0.26/0.67/1.95	1.41/0.69/0.25/0.09	3.6 ± 0.1 95 ± 35	
11		0.5+0.5/3(G)/3	21.0/7.3/4.7/4.1	34.2/6.4/1.3/0.4	0.19/0.36/0.80/2.07	1.63/0.88/0.28/0.10	5.3 ± 0.1 ^c^ 20 ± 5	
12	ZrS4(2-2) + Zr-Vin(2-2)	0.5+0.5/3(G)/2	25.1/8.0/4.8/4.0	46.2/8.6/1.7/0.5	0.26/0.49/1.03/2.64	1.84/1.07/0.36/0.12	6.8 ± 0.3 ^c^ 21 ± 2	Transparent, yellowish, homogeneous
13		0.25+0.25/3(G)/3	42.0/10.7/6.0/4.6	92.9/16.1/2.9/0.8	0.52/0.92/1.71/4.51	2.22/1.52/0.48/0.17	5.6 ± 0.1 ^d^ 41 ± 3	
14 ^a b^		0.25+0.25/3(G)/3	38.4/11.7/6.2/4.6	63.0/13.3/2.8/0.9	0.35/0.75/1.69/5.01	1.64/1.14/0.45/0.17	3.5 ± 0.1 66 ± 8	
15		0.25+0.25/3(E)/3	109.5/30.1/10.1/5.8	261.5/43.7/9.7/2.3	1.45/2.48/5.81/12.6	2.39/1.45/0.96/0.38	5.1 ± 0.3 ^c^ 12 ± 2	
16 ^a b^		0.25+0.25/3(E)/3	66.2/21.7/10.0/6.3	125.4/24.0/5.7/2.1	0.70/1.36/3.43/11.70	1.89/1.11/0.58/0.33	4.1 ± 0.2 195 ± 18	Opalescent, yellowish, homogeneous
17	ZrSU(2-2) + Zr-Ph(2-2)	0.5+0.5/3(G)/2	14.1/5.8/4.2/3.8	21.7/3.8/0.8/0.2	0.12/0.21/0.49/1.26	1.54/0.66/0.20/0.06	6.5 ± 0.1 ^d^ 119 ± 4	Transparent, yellowish, homogeneous
18		0.25+0.25/3(G)/3	21.1/7.9/4.7/3.9	32.0/7.1/1.5/0.4	0.18/0.40/0.91/2.30	1.51/0.90/0.32/0.10	6.3 ± 0.1 ^c^ 18 ± 7	
23	ZrSU(2-2) + Zr-Vin(2-2)	0.5+0.5/3(G)/2	19.2/6.3/4.2/3.7	24.7/5.1/1.1/0.3	0.14/0.29/0.63/1.55	1.28/0.81/0.25/0.07	6.7 ± 0.1 ^d^ 63 ± 14	Transparent, yellowish, homogeneous
24		0.25+0.25/3(G)/3	30.7/8.1/5.1/4.2	52.7/9.5/1.8/0.4	0.29/0.54/1.06/2.51	1.71/1.16/0.35/0.11	6.5 ± 0.2 ^c^ 21 ± 6	
25		0.25+0.25/3(A*)/3	8.1/5.2/4.1/3.8	3.5/1.5/0.5/0.15	0.02/0.09/0.28/0.83	0.44/0.29/0.11/0.04	4.3 ± 0.3 78 ± 10	Transparent, yellowish, homogeneous
26		0.25+0.25/3(E)/3	69.7/16.7/6.7/4.9	142.9/28.4/4.7/1.1	0.79/1.61/2.82/6.23	2.1/1.70/0.70/0.22	5.4 ± 0.1 ^d^ 34 ± 15	
27 ^b^		0.25+0.25/3(E)/3	31.9/14.1/8.2/5.4	89.2/14.2/3.6/1.5	0.50/0.80/2.12/8.47	2.80/1.00/0.43/0.27	2.2 ± 0.1 147 ± 9	Opalescent, yellowish, homogeneous
28	ZrSU(2-2) + ZrS4(2-2)	0.5+0.5/3(G)/2	10.5/5.3/4.1/3.8	17.5/2.9/0.6/0.2	0.10/0.17/0.38/0.96	1.67/0.54/0.15/0.04	6.5 ± 0.2 ^c^ 24 ± 4	Transparent, yellowish, homogeneous
29		0.25+0.25/3(G)/3	14.9/5.9/4.3/3.8	33.0/5.0/1.0/0.3	0.18/0.28/0.60/1.76	2.22/0.85/0.23/0.07	6.7 ± 0.2 16 ± 2	

* PDMS pre-blocked with 3-aminopropyltriethoxysilane; ε′—dielectric constant, ε″—dielectric losses, σ′—conductivity, tanδ—dielectric loss tangent, σ/ε—tensile strength/elongation at the moment of film rupture; ^a^ increase in the mixture viscosity with the MS addition; ^b^ accelerated composite drying; ^c^ rupture during the neck formation; ^d^ rupture during the neck spreading. The rubber brand is indicated in parentheses.

## Data Availability

The data presented in this study are available on request from the corresponding author.

## Supplementary Materials

The following supporting information can be downloaded at: https://www.mdpi.com/article/10.3390/polym15163361/s1, Figure S1: ^1^H NMR spectra of initial silanes 3 (a) and 4 (b) (in CDCl_3_) and the corresponding Rebrov’s salts, including after diffusion filtration (in toluene); Figure S2: ^1^H NMR spectra (a) of ZrS4(4-0), ZrS4(2-2) and ZrSU(2-2) (in CDCl_3_) and two-dimensional ^1^H-^29^Si HSQC correlation after diffuse filtration (b) for ZrS 4(2-2) toluene solution; Figure S3: Illustration of the nature of the stretching curves changing of compositions obtained with a combination of MSs, as well as with a change in the drying rate of the initial solution (with numerical values of elastic modulus E_o_); Table S1: Compositions characteristics obtained using ZrS4(1-3).
