# Supplementary material for: Thioether-Containing Zirconium(Alkoxy)Siloxanes: Synthesis and Study of Dielectric and Mechanical Properties of Silica-Filled Polydimethylsiloxane Compositions Cured by Them
Source: Polymers (Basel). 2023 Aug 10;15(16):3361. doi: 10.3390/polym15163361 (PMC10458246; doi:10.3390/polym15163361)
Supplement: Supplementary file 1 [file polymers-15-03361-s001.zip › polymers-2532717-supplementary.pdf]

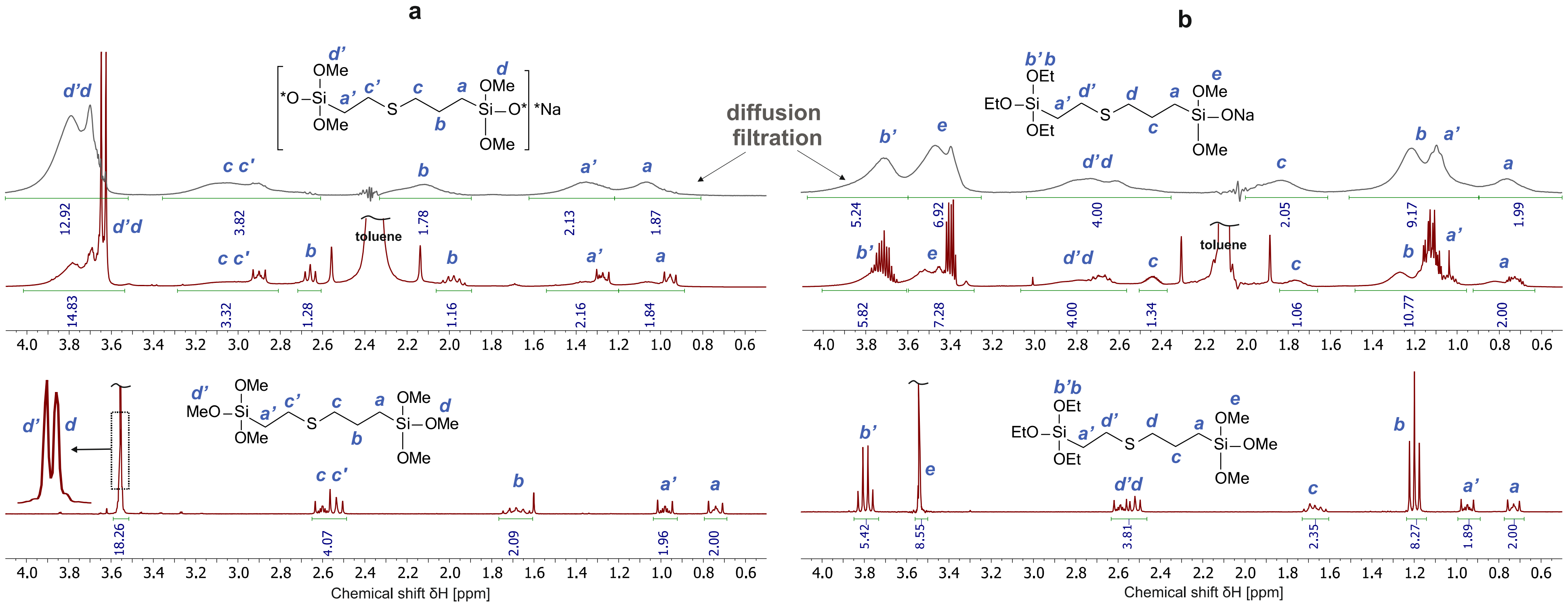

Figure S1. ^{1}H NMR spectra of initial silanes 3 (a) and 4 (b) (in $CDCl_3$) and the corresponding Rebrov's salts, including after diffusion filtration (in toluene).

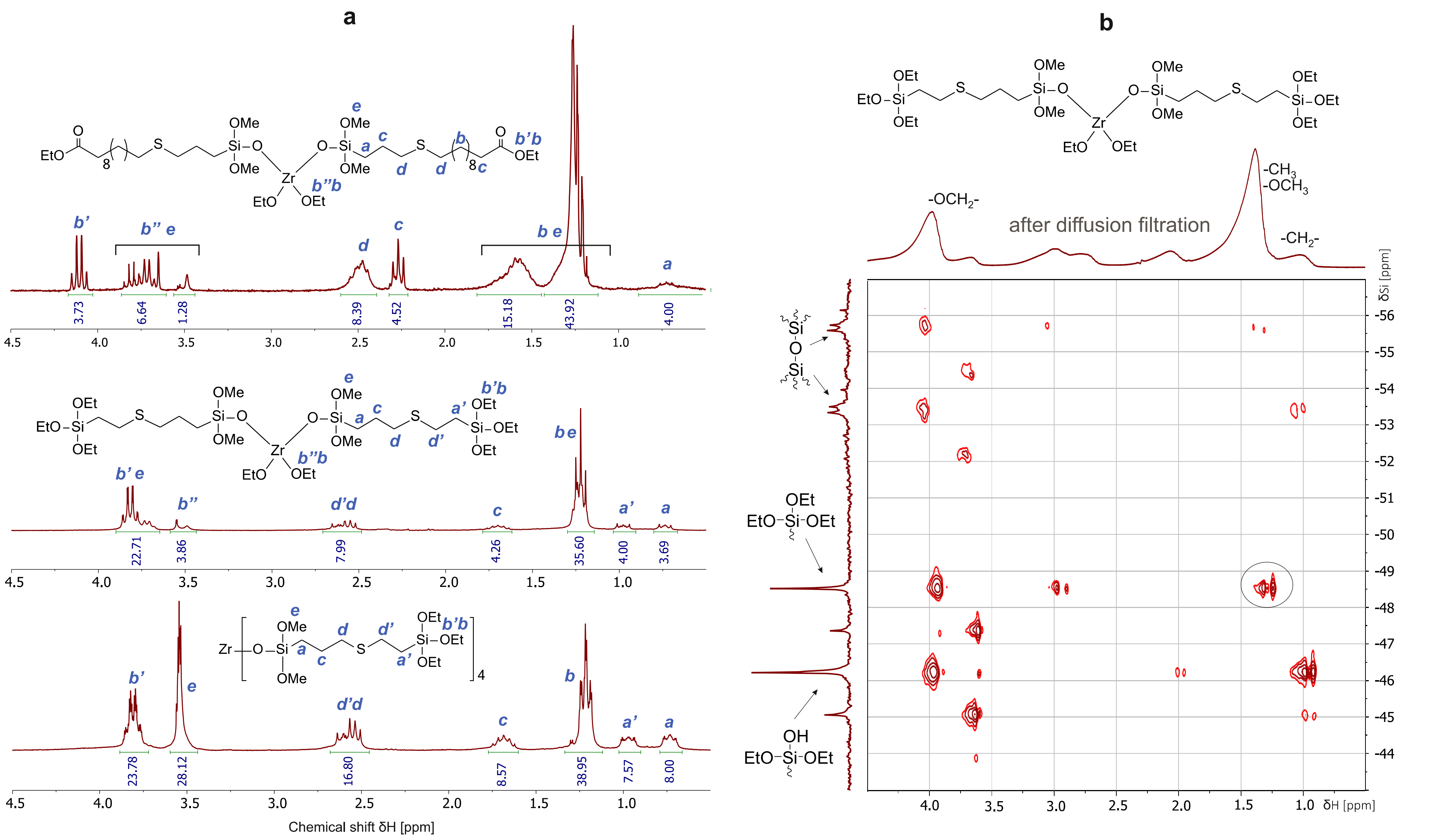

Figure S2. ^{1}H NMR spectra (a) of ZrS4(4-0), ZrS4(2-2) and ZrSU(2-2) (in $CDCl_3$) and two-dimensional ^1H-^{29}Si HSQC correlation after diffuse filtration (b) for ZrS 4(2-2) toluene solution.

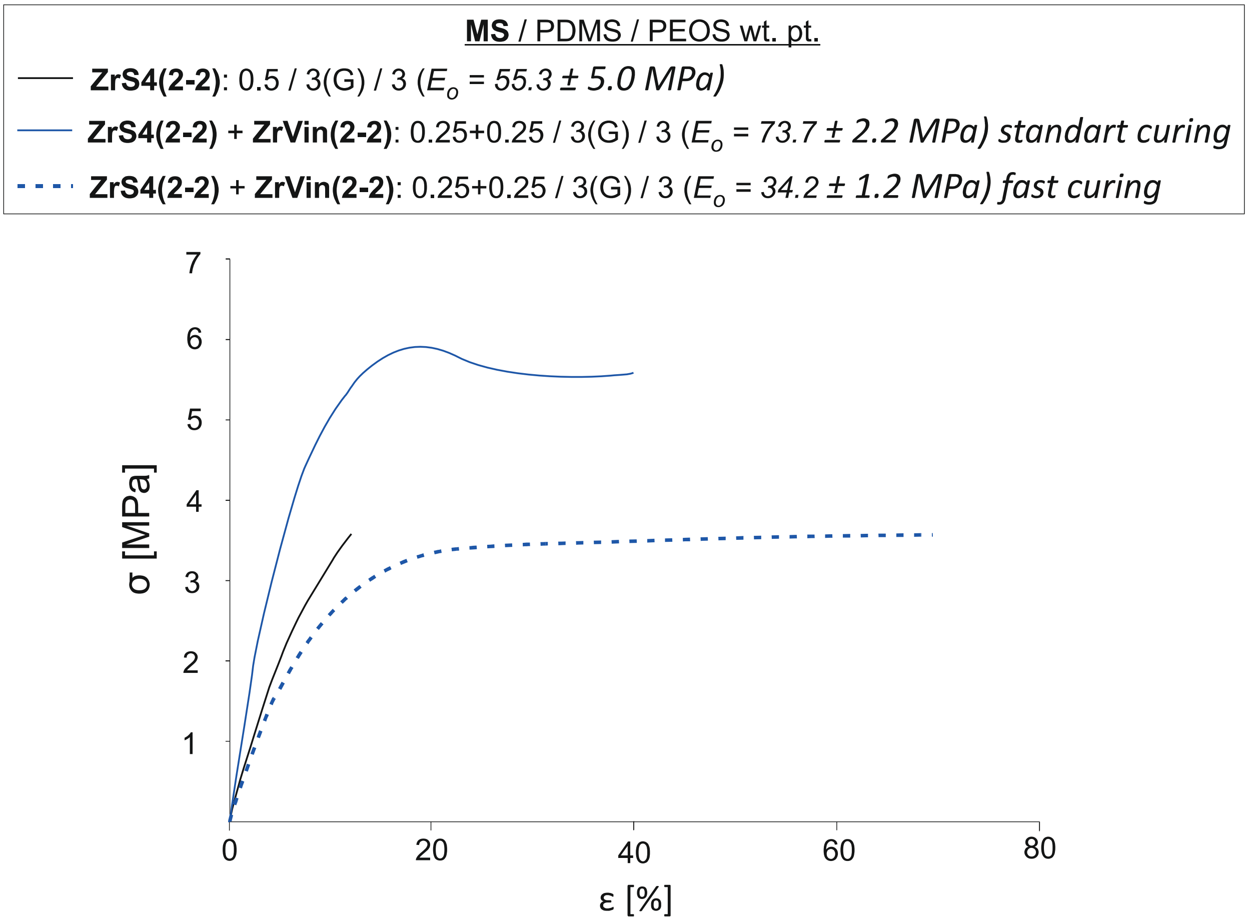

Figure S3. Illustration of the nature of the stretching curves changing of compositions obtained with a combination of MSs, as well as with a change in the drying rate of the initial solution (with numerical values of elastic modulus E_o).

Table S1. Compositions characteristics obtained using ZrS4(1-3).

#	Initial ratio ZrS4(1-3)/PDMS/PEOS wt.pt.	ε′	ε″	σ′·10[11] S/cm	tanδ	$\frac{\sigma \pm \Delta\sigma}{\varepsilon \pm \Delta\varepsilon}$ MPa/%	Characterization
		f = 0.1/1/10/100 Hz					
1	1 / 3(E) / -	3.2/2.9/2.8/2.7	0.8/0.2/0.1/0.02	0.005/0.01/0.04/0.17	0.25/0.07/0.04/0.01	3.4 ± 0.1 / 336 ± 34	Transparent, yellowish, homogeneous
2	1 / 3(E) / 1	13.5/6.1/4.2/3.6	27.7/4.6/1.1/0.3	0.15/0.26/0.65/1.74	2.05/0.75/0.26/0.08	5.1 ± 0.2 / 47 ± 15	
3	0.5 / 3(E) / 2	14.2/6.1/4.0/3.4	21.5/4.4/1.1/0.3	0.12/0.25/0.66/1.64	1.52/0.72/0.28/0.08	2.9 ± 0.1 / 48 ± 26	
4	1 / 3(E) / 2	21.6/8.0/4.6/3.8	25.9/6.9/1.6/0.4	0.14/0.39/0.96/2.41	1.20/0.86/0.35/0.12	4.8 ± 0.6 / 7 ± 1	
5 [a]	1 / 3(G) / 2	**25.9/12.1/5.7**/4.2	71.7/12.5/2.9/0.8	0.40/0.71/1.73/4.40	2.77/1/03/0.51/0.18	6.2 ± 0.1 [c] / 43 ± 4	
6 [a]	1 / 3(A) / 2	**38.8/12.3/5.7**/4.4	76.9/16.3/3.0/0.8	0.43/0.93/1.77/4.29	1.98/1.33/0.52/0.18	7.1 ± 0.3 / 27 ± 6	
7	1 / 3(G*) / 2	9.6/4.9/3.9/3.6	10.5/2.2/0.5/0.1	0.06/0.13/0.30/0.81	1.09/0.45/0.13/0.04	6.4 ± 0.3 / 13 ± 2	
8	0.5 / 3(E) / 3	19.3/7.4/4.4/3.7	41.4/7.8/1.6/0.4	0.23/0.44/0.96/2.18	2.15/1.04/0.36/0.10	3.9 ± 0.2 [c] / 13 ± 6	
9	2 / 3(E) / 2	24.1/9.8/4.8/3.8	40.0/10.0/2.1/0.5	0.22/0.56/0.12/2.90	1.66/1.01/0.43/0.14	0.3 ± 0.1 / 0.1 ± 0.1	Transparent, yellowish, homogeneous, brittle
10 [e]	1 / 3(E) / 2	10.7/4.8/3.6/3.3	10.6/2.4/0.6/0.2	0.06/0.14/0.33/0.90	0.99/0.51/0.16/0.05	8.2 ± 0.2 / 28 ± 2	Transparent, yellowish, homogeneous
11 [e]	0.5 / 3(E) / 2	12.3/6.7/4.4/3.8	13.6/3.3/1.0/0.3	0.08/0.19/0.61/1.58	1.11/0.50/0.23/0.07	3.0 ± 0.1 / 81 ± 55	

ε′ – dielectric constant, ε″- dielectric losses, σ′ - conductivity, tanδ - dielectric loss tangent, σ/ε - tensile strength/elongation at the moment of film rupture; * PDMS pre-blocked with 3-aminopropyltriethoxysilane; [a] increase in the mixture viscosity with the MS addition; [b] accelerated composite drying; [c] rupture during the neck formation; [d] rupture during the neck spreading; [e] previous study data obtained with Zr-Ph(1-3) using.
